# Foxm1 controls a pro-stemness microRNA network in neural stem cells

**DOI:** 10.1038/s41598-018-21876-y

**Published:** 2018-02-23

**Authors:** Zein Mersini Besharat, Luana Abballe, Francesco Cicconardi, Arjun Bhutkar, Luigi Grassi, Loredana Le Pera, Marta Moretti, Mauro Chinappi, Daniel D’Andrea, Angela Mastronuzzi, Alessandra Ianari, Alessandra Vacca, Enrico De Smaele, Franco Locatelli, Agnese Po, Evelina Miele, Elisabetta Ferretti

**Affiliations:** 1 Department of Experimental Medicine, Sapienza University, Rome, 00161 Italy; 2 Institute of Ecology, University of Innsbruck, Technikerstrasse 25, Innsbruck, a-6020 Austria; 3 David H. Koch Institute for Integrative Cancer Research, Massachusetts Institute of Technology, Cambridge, MA 02139 United States; 4 Department of Haematology, University of Cambridge, Cambridge Biomedical Campus, Long Road, Cambridge, CB2 0PT UK; 5 National Health Service Blood and Transplant, Cambridge Biomedical Campus, Long Road, Cambridge, CB2 0PT UK; 6 Center for Life NanoScience@Sapienza, Istituto Italiano di Tecnologia, Rome, 00161 Italy; 7 Department of Molecular Medicine, Sapienza University, Rome, 00161 Italy; 8 Department of Industrial Engineering, University of Rome Tor Vergata, Via del Politecnico 1, Rome, 00133 Italy; 9 Centre for Cell Signalling and Inflammation, Imperial College, London, SW72AZ UK; 10 Department of Pediatric Hematology-Oncology and Department of Neuroscience, Bambino Gesù Children’s Hospital, Rome, 00165 Italy; 11 Neuromed Institute, Pozzilli, 86077 Italy

## Abstract

Cerebellar neural stem cells (NSCs) require Hedgehog-Gli (Hh-Gli) signalling for their maintenance and Nanog expression for their self-renewal. To identify novel molecular features of this regulatory pathway, we used next-generation sequencing technology to profile mRNA and microRNA expression in cerebellar NSCs, before and after induced differentiation (Diff-NSCs). Genes with higher transcript levels in NSCs (vs. Diff-NSCs) included *Foxm1*, which proved to be directly regulated by Gli and Nanog. Foxm1 in turn regulated several microRNAs that were overexpressed in NSCs: miR-130b, miR-301a, and members of the miR-15~16 and miR-17~92 clusters and whose knockdown significantly impaired the neurosphere formation ability. Our results reveal a novel Hh-Gli-Nanog-driven Foxm1-microRNA network that controls the self-renewal capacity of NSCs.

## Introduction

Neural stem cells (NSCs) are a major focus of research owing to their capacity for self-renewal and their multipotency, both of which are determined by the expression of transcription factors and epigenetic regulations^1^. NSC biology is the result of a dynamic balance between the expansion and/or maintenance of an uncommitted stem cell pool and the processes of lineage restriction and differentiation^2,3^. Since the discovery of NSCs, researchers have been attempting to identify mechanisms underlying their maintenance, which might one day be exploited to repair tissue damage or improve the regenerative power of cells affected by neurodegenerative diseases (e.g., Parkinson’s disease, Huntington’s disease, multiple sclerosis) or spinal cord injury. A better understanding of the signalling cascades responsible for inducing proliferating NSCs to differentiate could therefore expand the potential uses of NSCs as therapeutic agents^4,5^.

The Hedgehog (Hh) signalling pathway plays fundamental roles in the maintenance of stem cells, including the NSCs of the cerebellum^3,6^. By the late stages of embryogenesis, Hh glycoproteins are being secreted by differentiated Purkinje neurons in the cerebellum^7^. These proteins bind to and inactivate the transmembrane receptor Patched on nearby target cells, abrogating its repression of a second transmembrane receptor, Smoothened. The complex downstream signalling triggered by these events culminates in the expression of glioma-associated oncogene (Gli)-family transcription factors (Gli1, Gli2, and Gli3), whose targets comprise genes promoting both the proliferation and survival of NSCs in the embryonic and adult brains^8^. Our previous studies showed that Gli1 and Gli2 in cerebellar NSCs directly regulate the pluripotency transcription factor Nanog^3^. In subsequent studies, Hh–Gli-driven expression of Nanog was shown to control a set of microRNAs (miRNAs) whose target genes encode proteins that inhibit the proliferation and diminish the self-renewal of these cells^9^.

In the present study, we used next-generation mRNA- and miRNA-sequencing to further explore this Hh–Gli-driven molecular network in early post-natal NSCs from the murine cerebellum. Our goal was to expand the map of this network by identifying other molecular players involved in the Hh-regulated self-renewal of these NSC pools.

## Results

### High-throughput transcriptome profiling of cerebellar NSCs

Cerebellar NSCs from postnatal day 4 (P4) mice were grown in stem-cell-selective medium, as described elsewhere^3^. As expected, under these conditions, the cells displayed high-level expression of stemness genes (*Nanog*, *Nestin)* and of *Gli1* (Supplementary Figure 1A). Transfer of these NSCs to differentiation medium^3^ was followed by significant increases in the expression of genes encoding astrocytic, neuronal, Purkinje, and oligodendrocytic cell markers (Supplementary Figure 1B).

**Figure 1:**
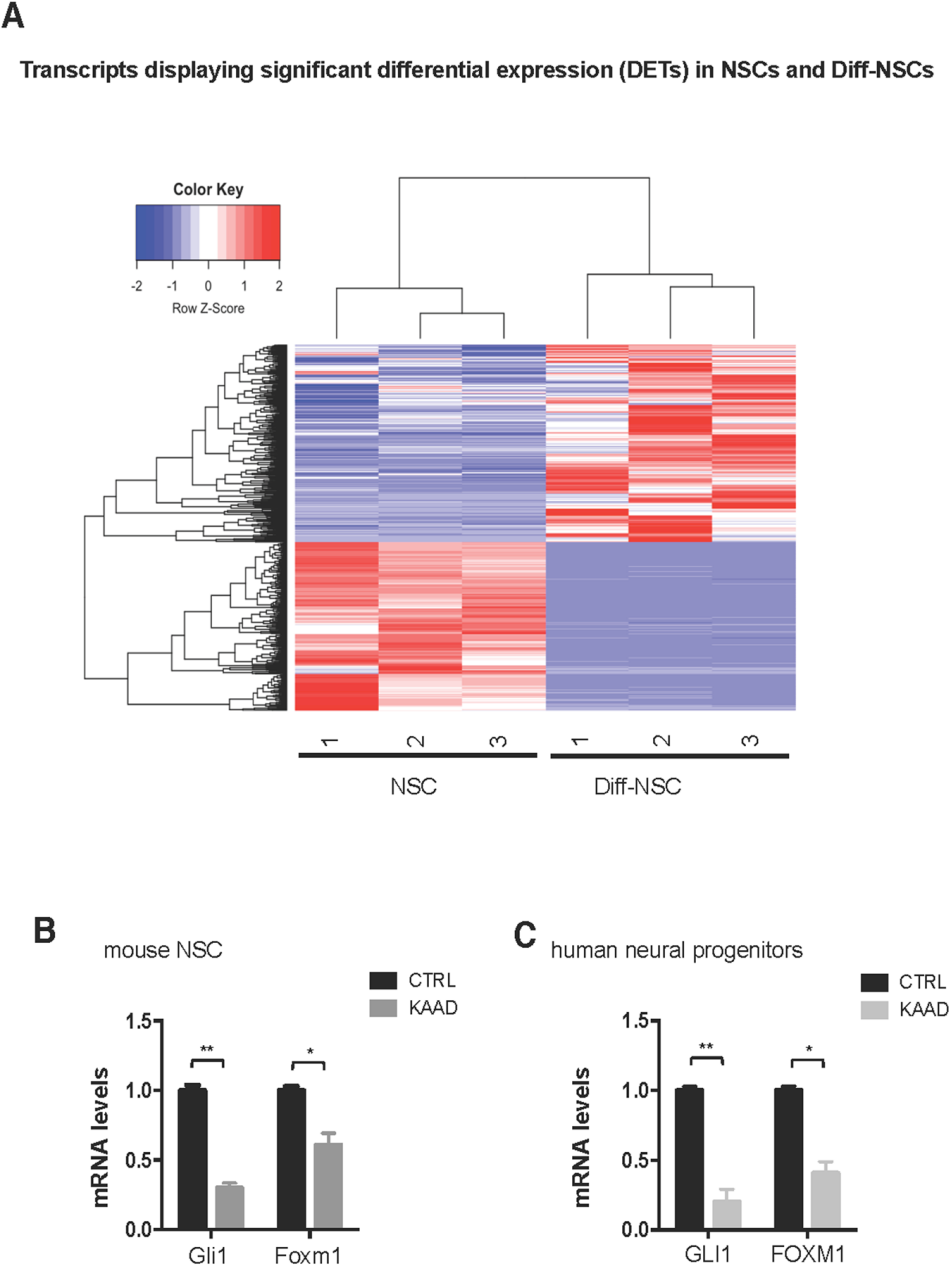
Foxm1 levels upon modulation of the Hedgehog pathway. (**A**) Hierarchical clustering of the 988 transcripts differentially expressed (adj. *P* < 0.05) (Bray-Curtis method with average linkage). (**B**) RT-qPCR data showing Gli1 and Foxm1 levels in mouse NSCs before and after 48 h of cyclopamine-KAAD (KAAD) treatment. *P* values vs. CTRL (DMSO as control) (*P < 0.05: 0.0412, **P < 0.01: 0.0044). (Mann–Whitney U test). (**C)** RT-qPCR data showing GLI1 and FOXM1 levels in normal human neural progenitors (NHNP) before and after 48 h of cyclopamine-KAAD (KAAD) treatment. *P* values vs. CTRL (DMSO as control). (*P < 0.05: 0.02, **P < 0.01: 0.0031). (Mann–Whitney U test). Bars in B and C represent the mean (SD) of three independent experiments.

Paired-end polyA^+^ RNA-sequencing was used to profile the transcriptomes of NSCs grown in stem cell and differentiation media (NSCs and Diff-NSCs, respectively; three replicates of each). A total of 988 genes were differentially transcribed by the cells under these two conditions. NSCs and Diff-NSCs were clearly segregated, as observed in the hierarchical clustering of the 988 differentially expressed transcripts (DETs) (Fig. 1A).

Functional analysis of the DETs using the DAVID platform (Database for Annotation, Visualization and Integrated Discovery) revealed significant enrichment (Bonferroni-corrected *P* < 0.05) for the four Gene Ontology categories reported in Table 1 and detailed in Supplementary Figure 2. The most interesting clue that emerged from this analysis was the over-representation of genes involved in p53 signalling. This pathway is a well-known negative regulator of NSC self-renewal^9,10^, whose activity is modulated by signalling through the Hh-Gli-Nanog axis^3,11^.

**Table 1:** Gene Ontology categories over-represented in the set of 988 DETs*.

Gene Ontology Category	Fold Enrichment	Count	%	Bonferroni-corrected *P*-value
Cell cycle	5.4	37	4	2.50E-15
DNA replication	8.1	15	1.6	1.50E-07
p53 signalling pathway	3.8	14	1.5	9.00E-03
ECM-receptor interaction	3.4	15	1.6	1.70E-02

*Findings are ranked according to Bonferroni-corrected *P* values.

### Hedgehog–Gli pathway components enriched in NSCs

To identify other molecular players with potential roles in Hh-Gli-driven self-renewal of cerebellar NSCs, we compiled a list of 53 genes known to be regulated by Hh-Gli signalling in settings (physiologic or pathologic) other than NSCs (Supplementary Table 1). Nine of these genes were differentially transcribed in NSCs before and after differentiation (Supplementary Table 2, Supplementary Figure 3A). Six of the nine genes encode cyclins (Ccnb2, Ccnb1, Ccna2, Ccnd2, Ccne1, Ccnd1) known to be involved in the regulation of cell cycle and cell division in NSCs. The seventh, *Sema6a*, is involved in nervous system development, in particular, in axon guidance^12^, and the eighth gene, *Insm1*, has been reported to be involved in mouse cerebellar development^13^.

**Figure 2:**
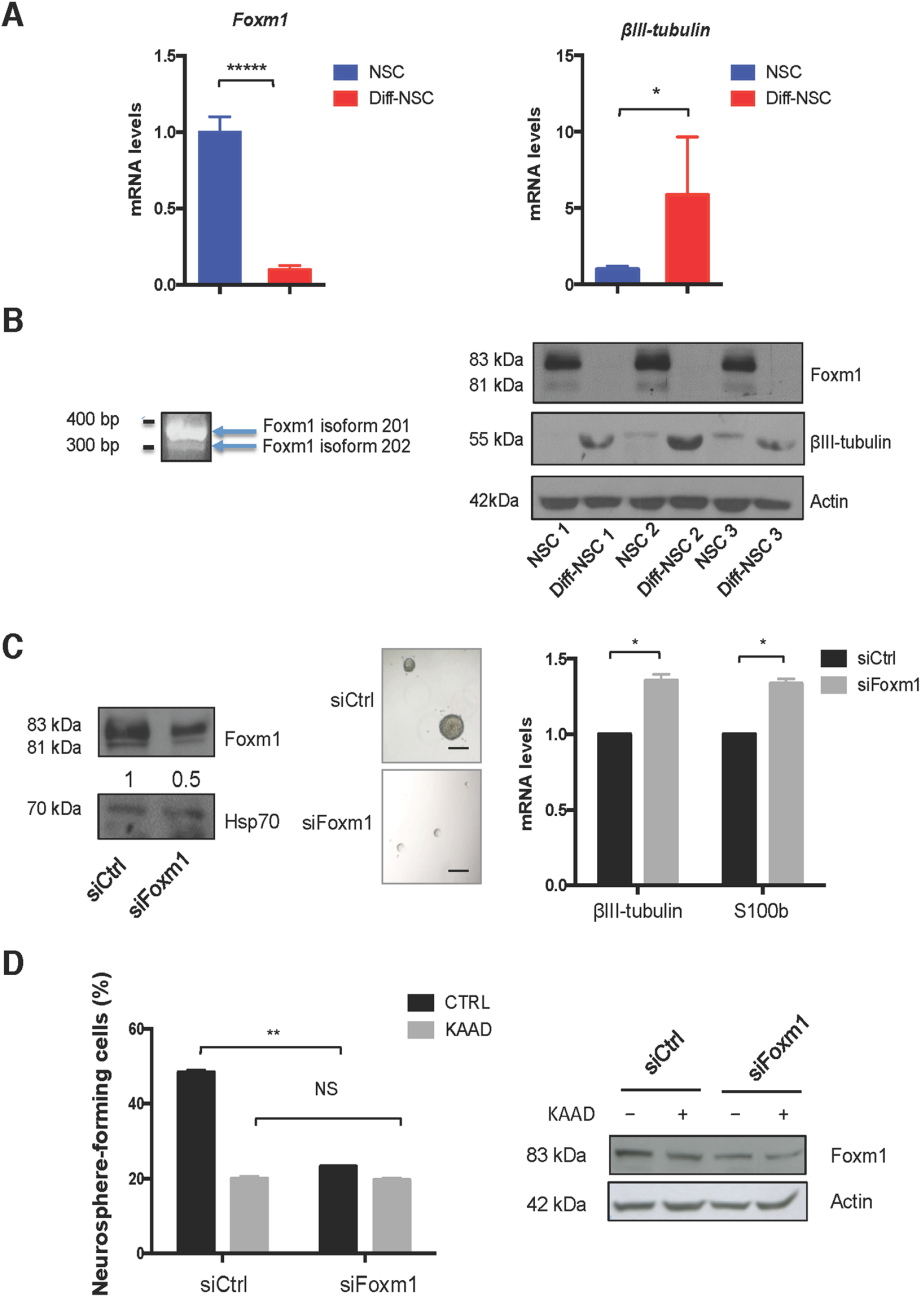
Upregulated expression of Foxm1 in P4 cerebellar NSCs and its effect on self-renewal. (**A**) RT-qPCR data showing differential expression in pre- and post-differentiation NSCs of mRNA for *Foxm*1 (*****P < 0.0001: 0.0000089) and the neuronal differentiation gene *β*III-tubulin (*P < 0.05: 0.029) (Mann–Whitney U test). (**B**) *Left*: PCR assay of *Foxm1* expression using isoform-specific primers. Agarose (2%) gel separation of the amplified product yielded two bands corresponding to *Foxm1* isoforms 201 (400 bp) and 202 (300 bp). Full-length gel is presented in Supplementary Figure 8A. *Right*: Immunoblots showing endogenous levels of Foxm1, *β*III-tubulin, and Actin (loading control) in three NSC cultures before and after differentiation. Full-length immunoblots are presented in Supplementary Figure 8B. (**C**) *Left:* Immunoblots showing endogenous levels of Foxm1 and Hsp70 (loading control) in NSCs transfected with siRNA against Foxm1 or non-targeting siRNA controls (siCtrl). Densitometric values appear below blots. Full-length immunoblots are presented in Supplementary Figure 8C. *Middle:* Representative bright-field images of neurospheres formed by NSCs transfected with siCtrl and siFoxm1. Scale bar: 100 μm. *Right:* RT-qPCR data showing mRNA levels of *β*III-tubulin (*P < 0.05: 0.043) and S100b (*P < 0.05: 0.031) in NSCs transfected with siCtrl and siFoxm1. *P* values vs siCtrl. (Mann–Whitney U test). (**D**) *Left:* Neurosphere-formation capacity of NSCs transfected with siCtrl and siFoxm1 and treated with cyclopamine-KAAD (KAAD) to suppress endogenous Hedgehog signalling. Graphs show percentage of seeded cells that formed neurospheres. *P* values vs siCtrl. (**P < 0.01: 0.003, NS: Not significant 0.072) (Two-way ANOVA test). *Right:* Immunoblots showing endogenous levels of Foxm1 in NSCs transfected with siCtrl and siFoxm1 and treated with cyclopamine-KAAD (KAAD). Full-length immunoblots are presented in Supplementary Figure 9A. Bars in panels A, C, and D represent the mean (SD) of three independent experiments.

Analysis of this list revealed that Foxm1 was the Hh-Gli-regulated transcription factor most markedly expressed in NSCs prior to differentiation. Foxm1 is a transcriptional activator^14^ whose role as a downstream mediator of Hh-Gli signalling has thus far been documented exclusively in human cancer cells^15–18^. The relation between Foxm1 and Hh-Gli signalling was also evident in our murine cerebellar NSC model. As shown in Supplementary Figure 3B,C, mRNA and protein levels in our NSCs displayed good correlation with Hedgehog pathway activation, which is known to peak in these cells on post-natal days 4–6^7,19^. In addition, suppression of Hh signalling with a Smoothened inhibitor cyclopamine-KAAD (KAAD) significantly diminished *Foxm1* mRNA levels in P4 cerebellar NSC (Fig. 1B), and similar effects were observed in human neural progenitors (NHNP) (Fig. 1C). Consistent with their high-level expression of *Gli1* (Supplementary Figure 1A), the NSCs displayed strikingly higher levels of Foxm1—at both the transcriptional (Fig. 2A) and protein levels (Supplementary Figure 3C)—prior to their differentiation. Review of our mRNA-seq data confirmed this NSC-associated upregulation for two of the four known protein-coding Foxm1 transcript isoforms (ENSMUST00000073316 [Foxm1-201] and ENSMUST00000112148 [Foxm1-202]) (Supplementary Table 3). As shown in Fig. 2B, this finding was validated by PCR performed with isoform-specific primers and by immunoblot analysis, which revealed clear predominance in the NSCs of the 757-amino-acid Foxm1-201 protein isoform.

### Foxm1 mediates Hh–Gli-driven self-renewal of the NSCs

**Figure 3:**
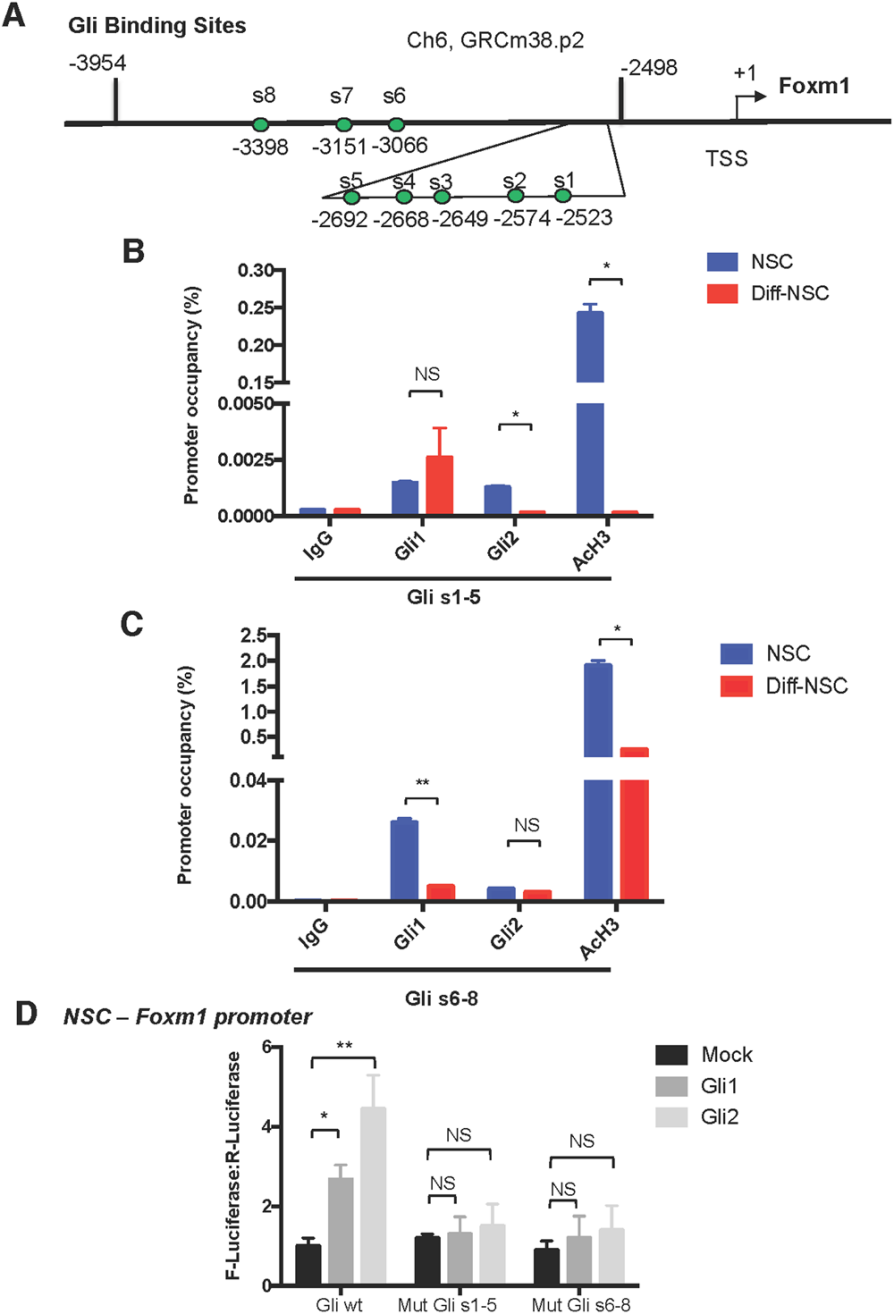
Foxm1 promoter occupancy by Gli1 and Gli2. (**A**) Schematic of the *Foxm1* promoter showing locations of the 8 putative Gli-responsive elements (s1–s8). (**B**,**C)** qPCR-ChIP assay of endogenous Gli1 and Gli2 occupancy of the *Foxm1* promoter region in NSCs and Diff-NSCs. Immunoprecipitation with IgG was performed as control. Anti-acetyl-H3 antibodies was used to detect Foxm1 transcriptional activation. Eluted DNA was qPCR-amplified using primers encompassing putative Gli binding sites [s1–s5 (**B**) and s6–s8 **(C)**]. Results are expressed as fold induction values relative to ChIP input controls. B-actin was utilized as unrelated chromatin control and is presented in Supplementary Figure 5 A. Bars represent means (SD) of three independent experiments. *P* values vs. Diff-NSCs (Mann-Whitney U test): (**B**) *P < 0.05: 0.04797 (s1-5, Gli2), 0.03271 (s1-5, AcH3); NS (not significant): 0.2514 (s1-5, Gli1). (**C**) *P < 0.05: 0.0490 (s6-8, AcH3); **P < 0.01: 0.001374 (s6-8, Gli1); NS: 0.296763205 (s6-8, Gli2). (**D**) Luciferase activity induced in the *Foxm1* promoter region in NSCs by Gli1, Gli2, and Mock (negative control, PCDNA). Results are normalized to pRL-CMV-Renilla luciferase (R-Luciferase). Bars represent means (SD) of at least three independent experiments, each performed in triplicate. *P* values vs. control cells (One-way ANOVA test): *P < 0.05: 0.02 (Gli wt-Gli1); **P < 0.01: 0.005 (Gli wt-Gli2), NS: Not significant 0.072 (Mut Gli s1-5-Gli1); 0.066(Mut Gli s1-5-Gli2); 0.083 (Mut Gli s6-8-Gli1); 0.077(Mut Gli s6-8-Gli2).

To explore the functional relevance of this upregulation, we transfected NSCs with siRNA directed against Foxm1 (siFoxm1) and evaluated their self-renewal capacity, as reflected by their ability to form neurospheres. As shown in Figs. 2C,D, *Foxm1* knockdown was associated with significantly impaired neurosphere formation. Interestingly, when we prevented Hh activation with the use of cyclopamine-KAAD (KAAD), (Fig. 2D) the silencing of Foxm1 did not exert any additional effect. We then investigated whether the knockdown of Foxm1 had an effect on differentiation, proliferation and apoptosis. As shown in Fig. 2C, the silencing of Foxm1 led to the up-regulation of differentiation markers *β*III-tubulin and S100b. We did not observe any significant modulations in proliferation and apoptosis markers (Supplementary Figure 4). Comparison of the human *FOXM1* and murine *Foxm1* promoter regions revealed a high percentage of identical base pairs (34–46%), which indicated substantial similarity (Supplementary Table 4). Consistent with recent findings on its human ortholog^17,18^, the murine *Foxm1* promoter was found to harbour eight putative Gli-binding sites (s1–s8) (Fig. 3A and Supplementary Information). We performed quantitative PCR-ChIP assays in order to determine whether both transcriptional activators of the Hh-Gli pathway could occupy the Foxm1 promoter in these putative binding sites. Experiments were performed in NSCs both before and after differentiation to quantitatively assess Gli recruitment and histone H3 acetylation (AcH3, a marker of transcriptional activation). Promoter occupancy was reported in all putative Gli binding sites (s1–5) and (s6–8) as evidenced by the higher percentage in NSCs when compared to Diff-NSCs. In particular, Gli2 reported a significantly higher percentage of occupancy in the Gli (s1–5) binding sites of the Foxm1 promoter in NSCs in respect to Diff-NSCs (Fig. 3B). Similarly, Gli1 promoter occupancy in the Gli (s6-8) binding sites of the Foxm1 promoter was significantly higher in NSsC when compared to Diff-NSCs (Fig. 3C**)**. No binding was observed in an unrelated chromatin region (Supplementary Figure 5A). Our data allow us to conclude that Gli-binding sites were bound by both transcriptional activators of the Hh-Gli pathway in NSCs.

Luciferase reporter assays showed significant activation of Foxm1-reporter by Gli2 and Gli1 binding both in 293 T and in the more physiological context of NSCs (Supplementary Figure 5B and Fig. 3D). Collectively, these findings strongly support the importance of Foxm1 as a major mediator of Hh–Gli-driven self-renewal of the NSC phenotype in the post-natal murine cerebellum.

### Foxm1 modulates stemness through the activation of specific miRNAs in NSCs

**Figure 4:**
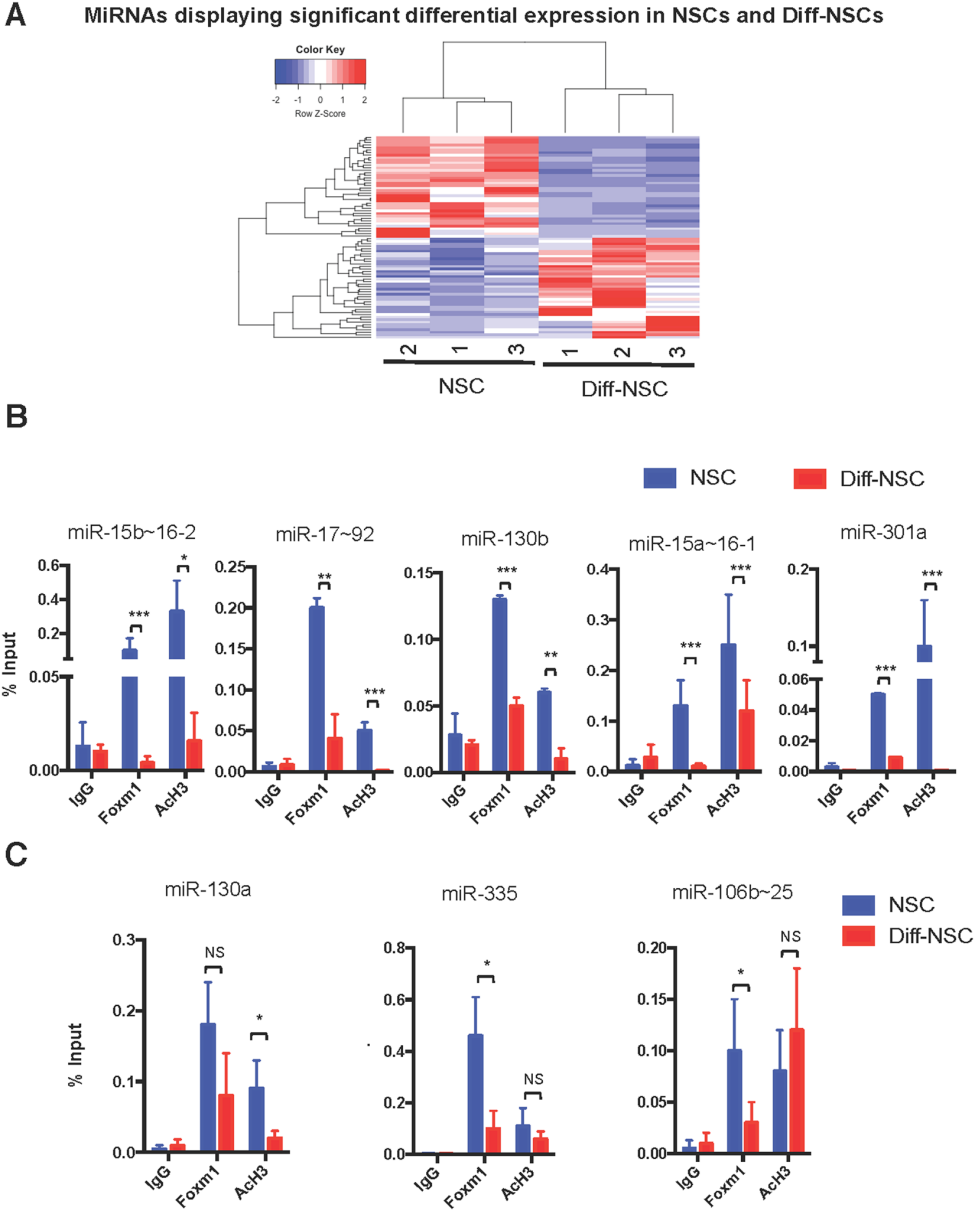
Foxm1 controls the transcription in P4 murine cerebellar NSCs of multiple miRNAs and miRNA clusters. (**A**) Heat map and dendrogram depiction of the 80 miRNAs displaying significant differential expression in NSCs before and after induction of differentiation. (**B**,**C**) qPCR-ChIP assays of NSCs and Diff-NSCs using anti-Foxm1 antibody and anti-acetyl-H3 antibody. Immunoprecipitation with IgG was performed as control. Eluted DNA was PCR-amplified with primers annealing to promoter regions of the miRNA genes of interest. Findings for miRNA candidates belonging to a cluster are based on assays of one representative cluster member. Results are expressed as fold induction versus input controls. B-actin was utilized as unrelated chromatin control and is presented in Supplementary Figure 6B. Bars represent the mean (SD) of three independent experiments. *P* values NSCs vs. Diff-NSCs (Mann–Whitney U test): Statistically significant (**B**) Foxm1: **P < 0.01: 0.002204 (miR-17~92); ***P < 0.001: 0.000 (miR-15b~16-2), 0.0003471 (miR-130b), 0.00004 (miR-15a~16-1), 0.0003906 (miR-301a). AcH3: *P < 0.05: 0.049416827 (miR-15b~16-2); **P < 0.01: 0.008868 (miR-130b); ***P < 0.001: 0.0008656 (miR-17~92), 0.00000069 (miR-15a~16-1), 0.00000920 (miR-301a). (**C**) Foxm1: *P < 0.05: 0.01467 (miR-335), 0.01021 (miR-106b~25); NS: not significant: 0.07214 (miR-130a). AcH3: *P < 0.05: 0.02903 (miR-130a); NS: 0.5259 (miR-335), 0.4417 (miR-106b~25).

As previously noted, the Hh-Gli-regulated stemness marker, Nanog, modulates the proliferation and self-renewal of murine cerebellar NSCs via miRNA-mediated suppression of genes promoting cell-cycle arrest and differentiation^3,9^. We wondered whether miRNAs might also play a role in Foxm1’s effects on NSC self-renewal. As shown in Fig. 4A, miRNA-sequencing studies identified 80 miRNAs that were differentially expressed in NSCs and Diff-NSCs.

To identify miRNAs likely to be direct targets of Foxm1, we examined the promoter regions of the 40 miRNAs that were upregulated in NSCs (Supplementary Table 5) and found putative Foxm1 binding sites in 20. To increase our chances of identifying targets with biological relevance to NSC self-renewal, we restricted our subsequent analyses to the 15 miRNAs on this list with the most statistically significant upregulated expression in the NSCs (Table 2, Supplementary Figure 6A). **(**See Supplementary Information file for the promoter regions of the miRNAs.)

**Table 2:** The miRNAs with putative Foxm1 binding sites that are most markedly upregulated in NSCs.

*No*.	MicroRNA	log_2_ fc*	adj. P-value	Member of microRNA Cluster	Part of microRNA Family	ChIP-confirmed
*1*	mmu-miR-15b-3p	2.75	2.33E-07	15b~16-2	15a, 15b, 16-1,16-2, 195a	Yes
*2*	mmu-miR-92a-1-5p	2.56	3.66E-04	17~92	25, 92a-1, 92a-2, 92b	Yes
*3*	mmu-miR-130b-5p	1.97	6.72E-04	—	130a, 130b, 301a, 301b	Yes
*4*	mmu-miR-130a-5p	1.98	1.54E-03	—	130a, 130b, 301a, 301b	No
*5*	mmu-miR-335-3p	2.66	2.27E-03	—	—	No
*6*	mmu-miR-25-5p	1.58	2.77E-03	106b~25	25, 92a-1, 92a-2, 92b	No
*7*	mmu-miR-93-3p	1.49	2.77E-03	106b~25	17, 18a,18b, 20a, 20b, 93, 106a, 106b	No
*8*	mmu-miR-15b-5p	1.6	3.88E-03	15b~16-2	15a, 15b, 16-1,16-2, 195a	Yes
*9*	mmu-miR-16-2-3p	1.71	7.46E-03	15b~16-2	15a, 15b, 16-1,16-2, 195a	Yes
*10*	mmu-miR-301a-5p	1.95	1.08E-02	—	130a, 130b, 301a, 301b	Yes
*11*	mmu-miR-130b-3p	1.69	1.14E-02	—	130a, 130b, 301a, 301b	Yes
*12*	mmu-miR-106b-5p	1.82	1.19E-02	106b~25	17, 18a,18b, 20a, 20b, 93, 106a, 106b	No
*13*	mmu-miR-16-1-3p	1.96	1.76E-02	15a~16-1	15a, 15b, 16-1,16-2, 195a	Yes
*14*	mmu-miR-15a-3p	1.48	1.89E-02	15a~16-1	15a, 15b, 16-1,16-2, 195a	Yes
*15*	mmu-miR-19a-3p	1.82	3.91E-02	17~92	17, 18a,18b, 20a, 20b, 93, 106a, 106b	Yes

*NSC vs. Diff-NSC.

Quantitative PCR-ChIP assays were then performed on NSCs before and after differentiation to quantitatively assess Foxm1 recruitment and histone H3 acetylation (AcH3) at the promoter region of each miRNA gene putatively targeted by Foxm1. The 15 miRNAs were transcribed singly or as part of a cluster **(**Table 2). In detail, in case of a cluster, for further experiments expression levels of one representative cluster member are reported. For all miRNAs tested, Foxm1 recruitment was higher in the NSCs that in the Diff-NSCs. We focused our attention on those miRNAs where promoter occupancy, as evidenced by AcH3, was significantly more intense in the NSCs (Fig. 4B,C). No binding was observed in an unrelated chromatin region (Supplementary Figure 6B). Foxm1 recruitment indeed was statistically significant for miR-130b, miR-301a, and miRNAs belonging to the miR-15~16 and miR-17~92 clusters (Fig. 4B).

**Figure 5:**
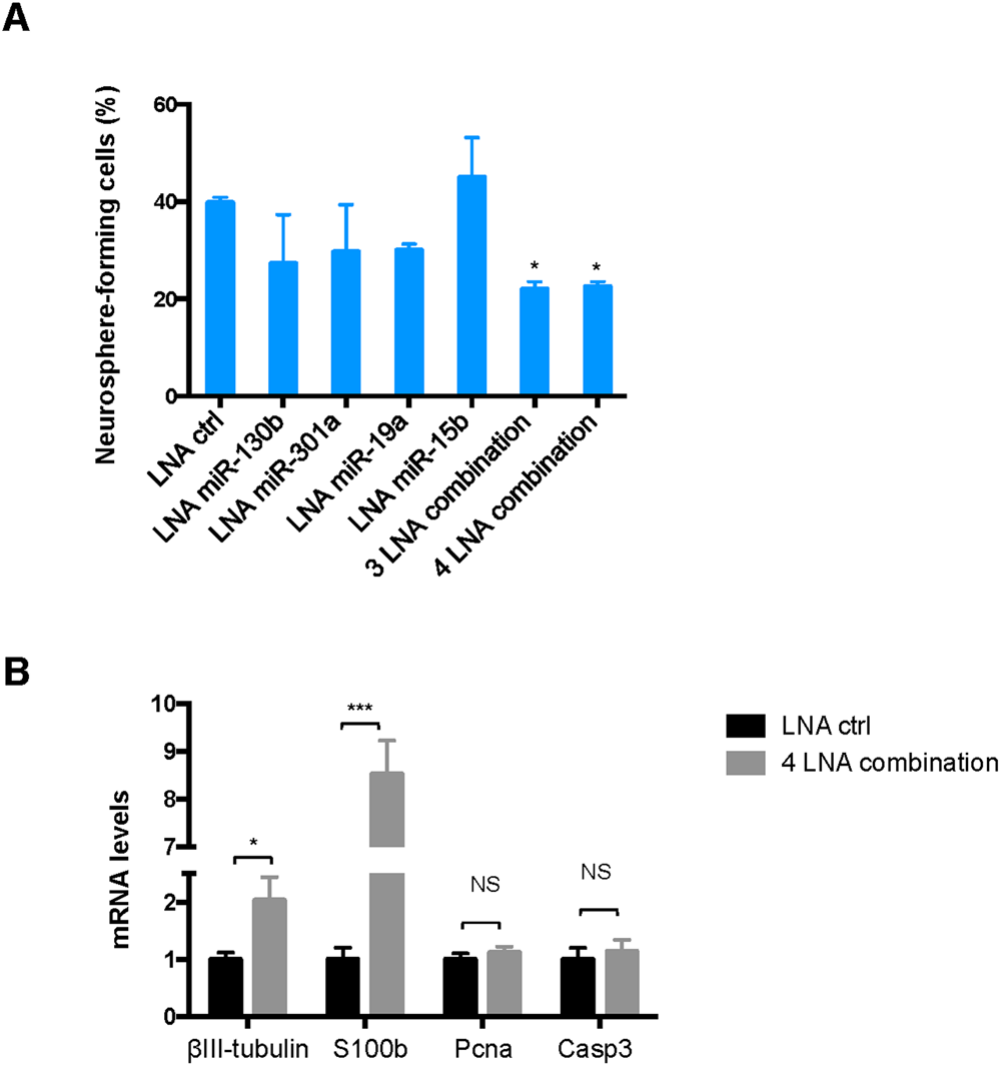
Foxm1-mediated miRNAs and miRNA clusters affect NSC neurosphere formation. (**A**) Neurosphere-formation capacity of NSCs transfected with LNA anti-miR-130b, -miR-301a, miR-19a (to inhibit miR-17-92 cluster members) and miR-15b (to inhibit miR-15–16 cluster members) that were used separately and combined. [3 LNA combination: anti- miR-130b, -miR-301a, and miR-19a; 4 LNA combination: anti-miR-130b, -miR-301a, miR-19a, and miR-15b]. *P* values vs. scrambled LNA control (One-way ANOVA test): *P < 0.05: 3 LNA combination: 0.0424; 4 LNA combination: 0.0500). (**B**) RT-qPCR data showing *β*III-tubulin, S100b, Pcna and Casp3 mRNA levels after 4 LNA combination. *P* values vs. scrambled LNA control (Two-way ANOVA test): *P < 0.05: 0.047 (*β*III-tubulin), ***P < 0.001: 0.00086 (S100b), NS: Not Significant (Pcna, Casp3). Bars in A and B panels represent means (SD) of at least three independent experiments.

The results of the previous experiments point to miR-130b, miR-301a, and miRNAs of miR-15~16 (n = 4) and miR-17~92 clusters (n = 2) as particularly important mediators of Foxm1’s effects in NSCs. Of note, expression levels of the above mentioned miRNAs were down-modulated after silencing of Foxm1 **(**Supplementary Figure 6C). Our claim was also supported by the effects observed in the cells after locked nucleic acid (LNA)-mediated depletion of these miRNAs. As shown in Fig. 5A, the NSCs’ capacity for neurosphere formation was not significantly reduced by anti-miR knockdown of any single miRNA or miRNA cluster. However, it was significantly impaired by combined depletion of miR-130b, miR-301a, and miR-19a (3 LNA combination), and the impairment was not significantly greater when miR-15b was also depleted (4 LNA combination). We investigated whether the knockdown of these miRNAs had an effect on differentiation, proliferation and apoptosis. As observed after the silencing of Foxm1 (Fig. 2C), also the knockdown of these downstream miRNAs resulted in the upregulation of differentiation markers, but had no effects on proliferation and apoptosis markers (Fig. 5B).

To identify possible mechanisms underlying the stemness-promoting effects of this miRNA network, we explored the genes targeted by the ChIP-confirmed miRNAs listed in Table 2. A miRTarBase (http://mirtarbase.mbc.nctu.edu.tw/) search returned validated murine targets for only three of these miRNAs: miR-15b-5p, miR-130b-3p, and miR-92a-3p (Supplementary Table 6). We therefore extended our search to the literature on each miRNA, focusing specifically on validated or putative target genes (in any species) whose downregulation could explain the combined effect of these miRNAs in NSC self-renewal.

The results that emerged reiterated the importance of p53 signalling, whose loss/suppression is essential for the maintenance of embryonic stem-cell pluripotency^20,21^. Of particular interest was a report showing that miR-130b-3p regulates CD133 + tumour-initiating cells in human hepatocellular carcinoma by targeting *TP53INP1*^22^, which encodes a downstream component of the p53 signalling pathway. Our previous work showed that Trp53inp1 expression in murine cerebellar NSCs is also suppressed by microRNAs of the miR-17~92 cluster, and the upregulated expression of these miRNAs was attributed to signalling through the Hh-Gli-Nanog axis^9^. In light of our current findings, p53 signalling in these cells also appears to be under the control of a second miRNA network, this one regulated by Foxm1. In support of this hypothesis, mirSVR prediction scores provided by microRNA.org (http://www.microrna.org/microrna/home.do) indicate that murine *Tp53inp1* is a likely target of miR-130b (mirSVR score: −0.0029). The same applies to miR-301a, another Foxm1-regulated microRNA belonging to the miR-130b family (mirSVR score: −0.0030). The Foxm1-regulated miRNA network that modulates p53 signalling might also comprise miR-92a-3p, whose validated targets include Trp63, another member of the p53 family of transcription factors. Because of the high-level sequence homology that characterizes these transcription factors, p63 and p73 are capable of transactivating p53-responsive genes, thereby causing cell cycle arrest and apoptosis^23^.

We cannot exclude of course other possible mechanisms of action for the Foxm1-mediated miRNAs. There are many ways in which these miRNAs can exert their function and an example is the report of high expression of miR-15a in MeCP2-deficient neural progenitors^24^, indicating its possible role in maintaining the undifferentiated state.

### A role for Nanog in Foxm1 regulation

**Figure 6:**
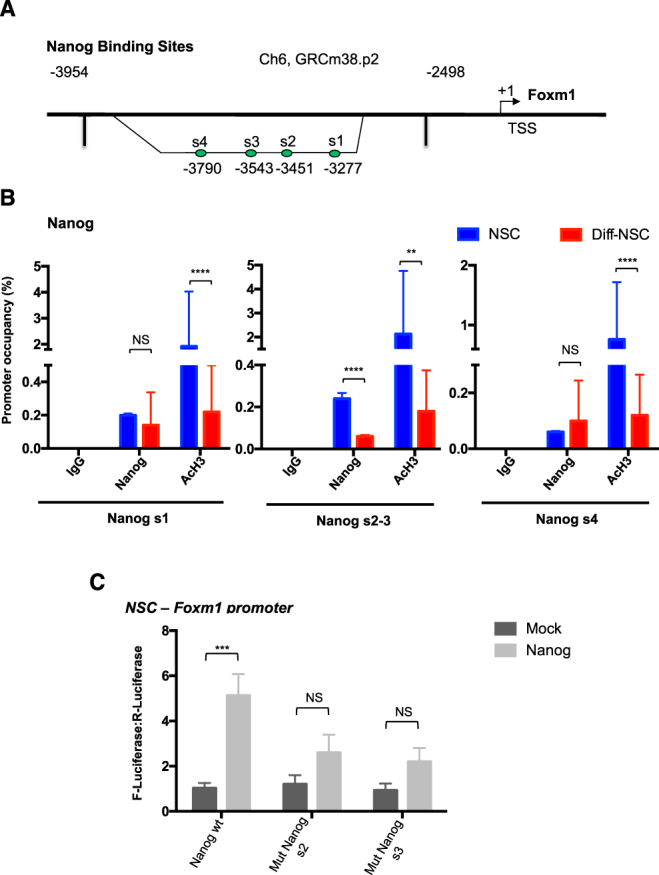
Foxm1 promoter occupancy by Nanog. (**A**) Schematic of the *Foxm1* promoter showing putative Nanog-responsive elements (s1; s2 - s3 and s4). (**B**) qPCR-ChIP assay of endogenous Nanog occupancy of the Foxm1 promoter region in NSCs and Diff-NSCs. Immunoprecipitation with IgG was performed as control. Anti-acetyl-H3 antibodies was used to identify Foxm1 transcriptional activation. Eluted DNA was PCR-amplified with primers for Nanog binding sites s1, s2-s3, s4. Results are expressed as fold induction values relative to input controls. B-actin was utilized as unrelated chromatin control and is presented in Supplementary Figure 7A. Bars represent means (SD) of three independent experiments. *P* values vs. Diff-NSCs (Mann-Whitney U test): **P < 0.01: 0.002572 (s2-3, AcH3); ****P < 0.0001: 0.00000718 (s2-3, Nanog), 0.0001051 (s1, AcH3), 0.00004356 (s4, AcH3); NS: 0.4531 (s1, Nanog), 0.7118 (s4, Nanog). (**C**) Luciferase activity induced by ectopic expression of Nanog and Mock (negative control, PCDNA) in NSCs transfected with luciferase vector carrying the wild-type Foxm1 promoter (wt) and its mutant lacking the Nanog binding sites s2 and s3 (mutants s2, s3). Bars represent means (SD) of at least three independent experiments, each performed in triplicate. *P* values vs. indicated controls (Mann–Whitney U test). ***P < 0.001: 0. 000937 (Nanog wt); NS: 0.097 (Mut Nanog s2), 0.18 (Mut Nanog s3).

Interestingly, members of the miR-17~92 cluster are components of both the Nanog-regulated^9^ and Foxm1-regulated miRNA networks, but this was the only commonality observed. This evidence indicates that Nanog and Foxm1 activate largely non-overlapping cohorts of miRNAs to ensure suppression of p53 signalling in cerebellar NSCs. How these two networks interact to achieve this goal is unclear. Interestingly, Nanog has been identified as a target of Foxm1^14,25,26^. To determine whether this relation might be reciprocal, we re-examined the Foxm1 promoter region for evidence of Nanog binding sites. As shown in Fig. 6A, four putative binding sites for Nanog were found −3790 to −3277 bp upstream from the Foxm1 TSS. (For details, see Supplementary Information).

ChIP experiments (Fig. 6B) demonstrated endogenous Nanog at these four sites in both NSCs and Diff-NSCs. However, pre- and post-differentiation occupancy rates were significantly different only at s2–s3, where the higher Nanog occupancy in NSCs was associated with greater activation of Foxm1. No binding was observed in an unrelated chromatin region (Supplementary Figure 7A). To further elucidate the relation between these two transcription factors, we performed dual luciferase reporter assays in NSCs transfected with Foxm1 wild-type promoter or an s2- or s3-defective mutant promoter (Fig. 6C). Ectopic expression of Nanog in these cells resulted in substantial activation of the Foxm1 wild-type promoter, whereas this induction was significantly reduced by deletion of critical nucleotides of s2 and s3, suggesting that these sites are required for Nanog binding onto the Foxm1 promoter. Interestingly, Foxm1 promoter luciferase reporter was significantly activated by Nanog also in 293 T cells (Supplementary Figure 7B).

Taken together, these results indicate that the Hh-Gli-driven miRNA networks regulated by Nanog and Foxm1 are characterized by bidirectional crosstalk, which might conceivably allow more finely tuned, combinatorial regulation of cerebellar NSC self-renewal.

## Discussion

**Figure 7:**
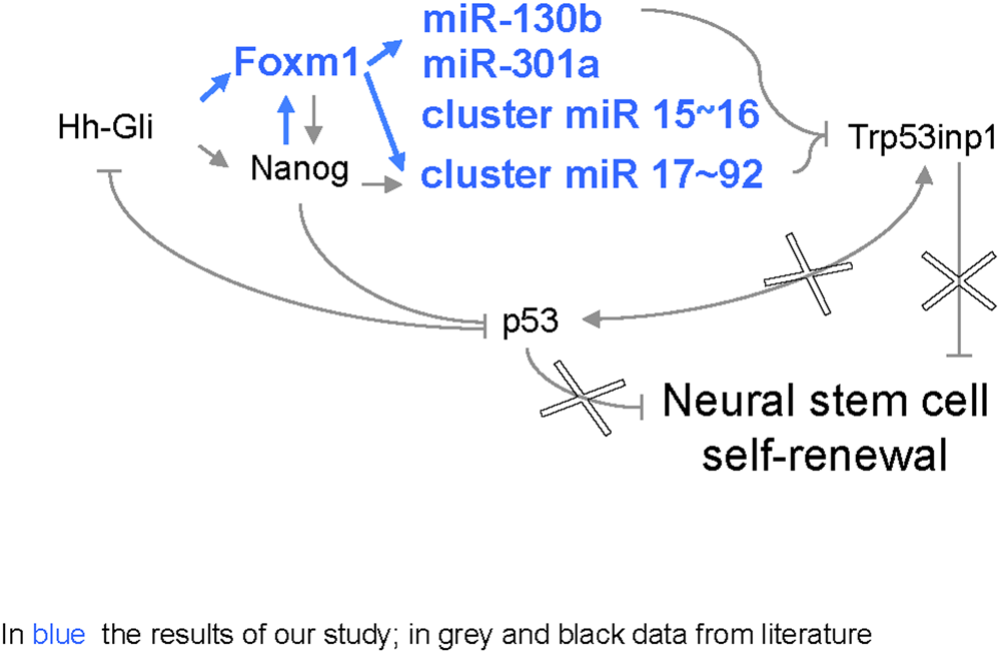
Regulation of cerebellar NSC self-renewal by the Hh-Foxm1-miRNA axis. Increased Hh-Gli signalling promotes NSC self-renewal by inhibiting p53 and upregulating Nanog expression. The tumour-suppressor p53 checks NSC self-renewal directly, by activating Trp53inp1, and by inhibiting Gli and Nanog. Our data show (bold-face type) that Hh-Gli signalling also upregulates the expression of Foxm1 (directly and indirectly via Nanog). The targets of these two transcription factors include a number of miRNAs that regulate the expression of important genes, including Trp53inp1. Repression of Trp53inp1 disrupts a feedback loop that maintains high p53 levels, thereby diminishing the tumour suppressor’s ability to repress Gli and Nanog expression.

The main goal of our study was to identify new molecular mechanisms involved in the Hh-Gli driven regulation of cerebellar NSC functions. As shown in Fig. 7, our findings indicate that Hh-Gli signalling controls self-renewal of murine NSCs in the P4 cerebellum via p53-pathway targeting miRNAs (i.e., those of the miR-17-92 cluster, that are also crucial for the expansion of cortical NSCs *in vivo*^27^), which are regulated not only by Nanog, as previously reported^9^, but also by a second transcriptional activator, Foxm1.

In human epidermal stem cells, Foxm1 has been shown to sustain the balance between self-renewal and commitment to terminal differentiation^28^. It also upregulates the expression of the neural stemness marker Nestin1 in NSCs from the embryonic cerebral cortices of mouse and is deemed critical for their self-renewal^14^. The results of experiments conducted with loss-of-function *Foxm1* mutants suggest that in the murine cerebellum, this transcription factor regulates cell cycling, mainly by adjusting the G2/M transition^9,29^, but no information has been published on Foxm1’s regulation or functions in cerebellar NSCs.

Our findings show that Foxm1 expression in these cells is controlled by Gli1 and Gli2, directly but also indirectly via their modulation of the expression of Nanog, which was shown to transcriptionally activate Foxm1. This dual mechanism of control is rendered even more complex by the fact that has been reported that Nanog is also a target of Foxm1^14,25,26^. As for the functions of Foxm1 in cerebellar NSCs, we also provide intriguing preliminary evidence suggesting that, aside from its above-mentioned co-regulation with Nanog of the miR-17-92 cluster, Foxm1 also controls other miRNAs (miR-130b and miR-301a) in these cells that putatively target the p53-signaling pathway. We used mRNA- and miRNA-sequencing to identify Foxm1-regulated miRNAs (other than those of the miR-17-92 cluster) with high-level expression in NSCs that drops significantly after these cells undergo differentiation. Knockdown of these miRNAs significantly impairs cerebellar NSC neurosphere formation by NSCs, suggesting these cells’ capacity for self-renewal is sustained by a Foxm1-regulated miRNA network.

Signals transmitted through the Hh-Gli-Foxm1 and Hh-Gli-Nanog axes clearly converge on miR-17~92 cluster members to suppress the expression of Trp53inp1. However, Trp53inp1 is also a putative target of two of the microRNAs regulated by Foxm1 alone, miR-130b^22^, which has already been associated with maintenance of embryonic NSCs^30^, and miR-301a. This observation suggests the possibility of combinatorial and partially redundant control of the p53 signalling cascade, which underlines the importance of this pathway in NSCs. Combinatorial repression of Trp53inp1, and consequently of p53, by both the Hh-Gli-Foxm1- and Hh-Gli-Nanog-driven miRNA networks, might be part of a hypothetical “bypass axis” that serves to maintain control of NSC self-renewal even in the presence of gene mutations affecting key components of the regulatory network. It is important to stress that the ability of miR-130b and miR-301a to downregulate Trp53inp1 expression requires verification in future studies. Nonetheless, self-renewal of the cerebellar NSCs was significantly reduced by combined knockdown of all four Foxm1-regulated miRNAs.

In conclusion, the regulatory network here proposed highlights the importance of Hh-Gli signaling and its downstream effector Foxm1 in cerebellar NSC maintenance and provides further insight into the complex mechanisms involved in the fine-tuning of stemness and differentiation cues in these cells.

## Methods

Unless otherwise specified, all commercial products were used in accordance with the manufacturer’s protocol.

### Cell models

#### Murine cerebellar NSC cultures

Cerebellar NSCs were isolated from P4 wild type black 6 /C56 (C57BL/6) mice (*n* = 8 per group) and cultured, as previously described^3^. In brief, freshly dissected cerebella were placed in HBSS supplemented with 0.5% glucose and penicillin-streptomycin and dissociated, mechanically and enzymatically. The cells were maintained as mycoplasma-free neurosphere cultures in selective stem-cell medium consisting of serum-free DMEM-F12 supplemented with 0.6% glucose, insulin 25 mg/ml, N-acetyl-L-cysteine 60 mg/ml, heparin 2 mg/ml, B27 supplement without vitamin A, EGF 20 ng/ml, and bFGF 20 ng/ml. To induce differentiation, NSCs were mechanically disaggregated and plated on poly-lysine coated support 48 h in differentiation medium consisting of the medium described above, prepared without the EGF/bFGF and supplemented with platelet-derived growth factor (PDGF; 10 ng/ml) (Sigma, P3076)^3^. Mycoplasma contamination was excluded by routine screening with the PCR Mycoplasma Detection Kit (ABM, Cat. No. G238). Animal experiments were performed according to the European Community Council Directive 2010/63/EU and were approved by the local Ethical Committee for Animal Experiments of the Sapienza University of Rome.

Normal Human Neural Progenitors (NHNP) (PT-2599) were obtained from ATCC (Milan, Italy) and cultured in selective stem-cell medium.

#### Treatments

NSCs and NHNPs were treated with a Smoothened antagonist for 48 h, cyclopamine-KAAD, (Calbiochem) at the concentration of 1 μM, DMSO was used as control.

### Experimental and analysis design

Please see Supplementary Information - Section 1. Supplementary Methods for a detailed description.

### mRNA- sequencing

Please see Supplementary Information - Section 1. Supplementary Methods for a detailed description of Library preparation and RNA sequencing.

### miRNA-sequencing

Please see Supplementary Information - Section 1. Supplementary Methods for a detailed description of MiRNA library preparation and sequencing.

### Bioinformatics analysis

All details regarding the mRNA-seq and miRNA-seq bioinformatics analysis can be found in Supplementary Information - Section 1. Supplementary Methods (Mapping of RNA-seq reads, mRNA differential expression analysis, functional analysis, clustering analysis, Mapping of miRNA-seq reads and miRNA differential expression analysis).

### Neurosphere-forming assay

Neurospheres grown in the stem-cell-selective medium were dissociated to single cell through dissociation solution non-enzymatic buffer (Cod: C5789, Sigma-Aldrich). Viable cells were counted after Trypan-blue exclusion and re-plated at clonal density (1-2 cells/mm²) in 96-well plates containing stem-cell selective medium. Results were expressed as the percentage of cells that gave rise to neurospheres.

### Immunofluorescence

Immunofluorescence experiments were performed using poly-lysine coated Labtek chamber slides as support: NSCs were allowed to adhere for three hours, while for differentiation studies, NSCs were dissociated and cultured as described in “*Murine cerebellar NSC cultures*”. Cells were fixed with 4% paraformaldehyde for 20 min at room temperature and incubated in blocking solution (5% normal goat serum (NGS), 1% BSA, 0.1% Triton X-100). Cells were incubated overnight with primary antibodies diluted in blocking solution and for 2 h with secondary antibodies. Primary antibodies were rabbit anti-Nanog polyclonal (Abcam ab80892); mouse anti-Nestin (Abcamab 11306); mouse anti-Gli1, #2643 (Cell Signaling Technology Inc); mouse anti-parvalbumin P3088 (Sigma); rabbit anti-S100 S2644 (Sigma); mouse anti-*β*III-tubulin (*β*IIItub) MAB1637 (Millipore); rabbit anti-Foxm1 (Santa Cruz Biotechnology, sc-502). Secondary antibodies (488-conjugated anti-mouse and anti-rabbit) were purchased from Molecular Probes (Invitrogen). Nuclei were Hoechst-counterstained and cover slips were mounted with fluorescence mounting medium (S3023) (Dako). Images were acquired with a Carl Zeiss microscope (Axio Observer Z1) using Apotome technology and AxioVision Digital Image Processing Software.

### Immunoblotting assay

Cells were lysed in Tris–HCl pH 7.6, 50 mM, deoxycholic acid sodium salt 0.5%, NaCl 140 mM, NP40 1%, EDTA 5 mM, NaF 100 mM, Na pyrophosphate 2 mM, and protease inhibitors. Lysates were separated on an 8% acrylamide gel and immunoblotted using standard procedures. Membranes were blocked for 1 h at room temperature in 5% nonfat dry milk and incubated overnight at 4 °C with the following antibodies: rabbit anti-Foxm1 (Santa Cruz Biotechnology, sc-502), rabbit anti-Hsp70, (Santa Cruz Biotechnology, sc-33575), mouse anti- *β*IIItub (MAB 1637 Millipore), goat anti-actin I-19 (sc-1616; Santa Cruz Biotechnology), anti-Nanog (PA1–41577, Thermo Fisher), anti-α-Tubulin Antibody (T9026 SIGMA). HRP-conjugated secondary antisera (Santa Cruz Biotechnology) were applied and binding visualized by enhanced chemiluminescence (ECL Amersham).

### RNA isolation and quantitative RT-PCR

RNA was isolated from cells, as previously described^31^. The High Capacity cDNA reverse transcription kit (Applied Biosystems Life Technologies, ThermoFisher) was used to synthesize cDNA, as previously described^32^.

Quantitative reverse transcription (RT–PCR) analysis was performed using a High Capacity cDNA Reverse Transcription kit. mRNA expression was analysed on cDNAs using the ViiA™ 7 Real-Time PCR System, SensiFAST™ Probe Lo-ROX (Bioline), TaqMan gene expression assay according to the manufacturer’s instructions (Applied Biosystems). Each amplification reaction was performed in triplicate, and the average of the three threshold cycles was used to calculate the amount of transcripts in the sample (SDS software, AB). mRNA quantification was expressed, in arbitrary units, as the ratio of the sample quantity to the calibrator or to the mean values of control samples. All values were normalized to three endogenous controls: *ß-2-microglobulin*, *Hprt and Gusb*.

mRNA expression was evaluated through TaqMan® Gene Expression Assays, using the following assays: Foxm1 (Mm00514924_m1), S100b (Mm00485897_m1), *β*IIItub (Mm00727586_s1), Pcna (Mm00448100_g1), Casp3 (Mm01195085_m1), ß-2-Microglobulin (Mm00437762_m1), Hprt (Mm03024075_m1) and Gusb (Mm01197698_m1).

mRNA of human samples was assessed using SensiFAST™ Sybr Lo-ROX (Bioline), with the following primers:

- Forward primer 5′-CTGCTTGCCAGAGTCCTTT-3′
- Reverse primer 5′-CTCCACCTGAGTTCTCGTCA-3′

MiRNA expression was assessed with Taqman probes, as previously described^33^, using the following miRNAs: miR-15b-3p (Code: 002173), miR-92a-1-5p (Code: 464504_mat), miR-130b-5p (Code: 002460), miR-130a-5p (Code: 462691_mat), miR-335-3p (Code: Mm03307393_pri), miR-25-5p (Code: PM12401), miR-93-3p (Code: PM12787), miR-15b-5p (Code: 000390), miR-16-2-3p (Code: 462713_mat), miR-301a-5p (Code: 006346_mat), miR-130b-3p (Code: 000456), miR-106b-5p (000442), miR-16-1-3p (Code: 002489), miR-15a-3p (Code: 002488), miR-19a-3p (Code: 000395).

### PCR for Foxm1 isoforms

cDNA from NSCs was used to discriminate Foxm1 isoforms. PCR was performed according to the manufacturer’s protocol (GoTaq® DNA Polymerase, Promega), using the following isoform-specific primers:

- Forward primer 5′-CAAGCCAGGCTGGAAGAACTC-3′
- Reverse primer 5′-GTTCACTGGGAACTGGATGGG-3′

PCR products were separated on 2% agarose gel.

### *In vivo* experiments

Cerebellum tissues were collected for analysis from CD1 wild-type mice at the age of 1, 4, 10, 15 and 22 days postnatal. Samples were evaluated for immunoblotting assays and RT-PCR analyses.

### Statistical analysis of *in vitro* experiments

Unless otherwise indicated, statistical analyses were performed with StatView 4.1 software (Abacus Concepts). The Mann–Whitney U test for unpaired data was used to analyse differences in gene expression between NSCs and Diff-NSCs and the unpaired T-test, paired T-test, one-way ANOVA and two-way ANOVA were used where appropriate. Results are expressed as means (S.D.) from at least three experiments.

### Identification and characterization of binding sites in Foxm1 and miRNA promoter regions

Please see Supplementary Information - Section 1. Supplementary Methods.

### Luciferase-reporter assays

293 T cells purchased from ATCC (Milan, Italy) and NSCs were used for luciferase-reporter assays. 293 T cells were seeded into 24-well plates and transfected 24 h later with mouse *Foxm1* promoter luciferase vector (1457 bp) (Genecoepia, Catalogue No.: MPRM26630-PG02, Gene Accession: NM_008021) with Lipofectamine® 2000 Reagent (Invitrogen). NSCs were transfected with mouse *Foxm1* promoter luciferase vector in 24-well plates with Fugene6 Transfection Reagent (Roche, Basel, Switzerland). For both cell models, the transfection included 80 ng of the *Foxm1* promoter luciferase vector, 400 ng of inductor (Nanog, Gli1, Gli2, or (as a negative control, Mock) PCDNA), and 2 ng of CMV-Renilla Luciferase control vector. Cells were harvested 24 h post-transfection and tested with the dual-luciferase assay (Promega). Results were expressed as firefly/Renilla luciferase activity ratios (F-luc/R-luc) and represent means (SD) of at least three experiments, each performed in triplicate. Foxm1 promoter luciferase vector was used to generate, by site-directed mutagenesis, the mutant derivatives lacking Nanog-binding sites.

### Site-directed mutagenesis

The QuickChange Multi Site-Directed Mutagenesis Kit (Agilent Technologies) was used to mutagenize Foxm1 promoter construct by deleting critical nucleotides in the putative identified binding sites. In detail, for Nanog-binding sites s2 and s3 and for Gli-binding sites s1-5 and s6-8. The reaction was carried out with the following primers:

- NBS2 Fw: CGGCTTTACTGTCCTAAGTCAAGAAAATAAACAAAGTTATCACAGGAG
- NBS3 Fw: GGTTTCCCTCTCTTCTGTTAACAGATCTTACACCGC
- GBS1 Fw: GTCGGCACTGCCGAGGGGGTGTTGGG
- GBS2 Fw: CGGCCTGTGAGGTGACGGCCGGG
- GBS3 Fw: CCCGCCCCGGGCTGGCCGTCC
- GBS4 Fw: GCCCGGGCTCCGCCCCGGGC
- GBS5 Fw: GCTGCGGGACCCGGGCTCCCCC
- GBS6 Fw: GCCAGAAACCCAAGCGCGTGGACTGAGC
- GBS7 Fw: CCGCCTCCTCCGCCCCCGAGGT
- GBS8 Fw: GCGGGAGGGCGAGCGAAGGCGGAAAC.

### Chromatin immunoprecipitation (qPCR-ChIP assay)

ChIP was performed using the MAGnify™ Chromatin Immunoprecipitation System (Invitrogen). Briefly, for each ChIP reactions 300000 cells were used, the cells were cross-linked 10 min with 1% formaldehyde and the reaction was stopped with 0.125 M glycine for 10 min at room temperature. Cells were washed and harvested, and membranes were lysed with Lysis Buffer and Protease Inhibitors (200×) for 5 min on ice. Chromatin was fragmented to obtain chromatin fragments of about 400–600 nucleotides. After sonication, the samples were spun at 10000 rpm at 4 °C for 10 minutes to pellet the cell debris. The primary antibody was coupled for 1 h to the protein A/G Dynabead, at the same time the lysate samples were diluted in Dilution Buffer/Protease Inhibitor (200×). After 1 h the lysates were added to their respective antibody/beads for 2 h. After which the lysates were washed three times with IP Buffer 1 and twice with IP Buffer 2. After the final washing, the cross-linking was reversed with Reverse Crosslinking Buffer and proteinase K at 55 °C for 15 minutes and then the DNA was purified and precipitated. Eluted DNA was PCR amplified with primers encompassing the Gli- and Nanog- responsive sites of murine Foxm1 promoter. The following antibodies were used: IgG rabbit (Invitrogen), rabbit polyclonal anti-Foxm1 C-20 (Santa Cruz Biotechnology, code: sc-502), rabbit polyclonal anti-Gli1 2553 (Cell Signaling), rabbit monoclonal anti-Nanog (Cell Signaling, code: 8600), rabbit polyclonal anti-Gli2 (Santa Cruz Biotechnology, code: sc-271786×), rabbit polyclonal anti-acetyl-histone 3 (Millipore, code: 06599). Eluted DNA has been analysed with Q-PCR and the B-actin gene was used as control. Primers were designed with Primer-Blast designing tool (http://www.ncbi.nlm.nih.gov/tools/primer-blast/)^34^ and Primers tool (Genomatix Genome Analyzer, GGA, v3.30126, https://www.genomatix.de/) and are reported in Supplementary Table 7.

### Knockdown studies

Small-interfering-RNA knockdown of Foxm1 was performed in NSCs with ON-TARGETplus SMARTpool (L-057933-01-0005), control ON-TARGETplus Non-targeting siRNA knockdown (D-001810-02-05) (Dharmacon). Hiperfect reagent (Qiagen) was used for transfections. Cells were harvested for assay 72 h post-transfection. Transfection efficiency was > 80%, monitored using the fluorescent control SiGLO (Dharmacon) and calculated as the percentage of fluorescent cells. All experiments were performed using the using the best knockdown efficiency without off-target effects, controlled using three different housekeeping genes (ß−2-microglobulin, Hprt and Gusb) for RNA studies and using Actin or Hsp70 for immunoblots.

Knockdown of miRNA expression was done with miRCURY LNA™ miRNA inhibitor (Exiqon, miR-130b-5p: code 4101000-011; miR-301a-5p: code 4101560-011, miR-15b-3p: code 4101249-011, miR-19a-3p: code 4101300-101), individually or in combination, and a scrambled control, referred to as LNA ctrl (Exiqon mirCURY knockdown probe control A: code 199002-08) (Exiqon). Hiperfect reagent (Qiagen, Hilden, Germany) was used to transfect the siRNA constructs into NSCs. The final concentrations were 10 nM (for single transfection) and 50 nM (for combination transfections). miRCURY LNA™ miRNA inhibitors are conjugated with fluorescent labels for monitoring of transfection efficiency and efficiency rate was 90%.

### Validated targets of miRNAs

The validated miRNA targets reported in Supplementary Table 6 were identified with miRTarBase (http://mirtarbase.mbc.nctu.edu.tw/)^35^, where each Foxm1-regulated miRNA was used as input.

### Putative miRNA target genes

MicroRNA.org (http://www.microrna.org/microrna/home.do) database was queried for putative miRNA target genes, where miRNA-target interactions were predicted with miRanda 3.3a and scores were calculated with mirSVR (good mirSVR score less than or equal to −0.1).

### Data availability

All data generated or analysed during this study are included in this published article (and its’ Supplementary Information files).

## Electronic supplementary material

Supplementary Figures and Information

Supplementary Tables
