# Supplementary material for: Foxm1 controls a pro-stemness microRNA network in neural stem cells
Source: Sci Rep. 2018 Feb 23;8:3523. doi: 10.1038/s41598-018-21876-y (PMC5824884; doi:10.1038/s41598-018-21876-y)
Supplement: Supplementary file 1 — Supplementary Figures and Information [file 41598_2018_21876_MOESM1_ESM.docx]

**Foxm1 controls a pro-stemness microRNA network in neural stem cells**

Zein Mersini Besharat^1,#^, Luana Abballe^1,#^, Francesco Cicconardi^2^, Arjun Bhutkar^3^, Luigi Grassi^4,5^, Loredana Le Pera^6^, Marta Moretti^7^, Mauro Chinappi^8^, Daniel D’Andrea^9^, Angela Mastronuzzi^10^, Alessandra Ianari^3^, Alessandra Vacca^1^, Enrico De Smaele^1^, Franco Locatelli^10^, Agnese Po^7,*^, Evelina Miele^6,10,$^ and Elisabetta Ferretti^1,11, $,*^

^1^ Department of Experimental Medicine, Sapienza University, Rome 00161, Italy

^2^ Institute of Ecology, University of Innsbruck, Technikerstrasse 25, Innsbruck a-6020, Austria.

^3^ David H. Koch Institute for Integrative Cancer Research, Massachusetts Institute of Technology, Cambridge, MA 02139, United States

^4^ Department of Haematology, University of Cambridge, Cambridge Biomedical Campus, Long Road, Cambridge CB2 0PT, UK

^5^ National Health Service Blood and Transplant, Cambridge Biomedical Campus, Long Road, Cambridge CB2 0PT, UK

^6^ Center for Life NanoScience@Sapienza, Istituto Italiano di Tecnologia, Rome 00161, Italy

^7^ Department of Molecular Medicine, Sapienza University, Rome 00161, Italy

^8^ Department of Industrial Engineering, University of Rome Tor Vergata, Via del Politecnico 1, Rome 00133, Italy

^9^ Centre for Cell Signalling and Inflammation, Imperial College, London SW72AZ, UK

^10^ Department of Pediatric Hematology-Oncology and Department of Neuroscience, Bambino Gesù Children’s Hospital, Rome 00165, Italy

^11^ Neuromed Institute, Pozzilli 86077, Italy

^#^ These authors contributed equally to this work.

^$^ These authors contributed equally to this work.

***Corresponding Authors:**

Elisabetta Ferretti, MD PhD

Department of Experimental Medicine - Sapienza University, Rome, Italy

Viale Regina Elena, 291 - 00161 Rome Italy; Phone (+39 06) 49255135 - Fax (+39 06) 49255660

E-mail: [elisabetta.ferretti@uniroma1.it](mailto:elisabetta.ferretti@uniroma1.it)

Agnese Po, PhD

Department of Molecular Medicine - Sapienza University, Rome, Italy

Viale Regina Elena, 291 - 00161 Rome Italy; Phone (+39 06) 49255133 - Fax (+39 06) 49255660

E-mail: [agnese.po@uniroma1.it](mailto:elisabetta.ferretti@uniroma1.it)

**Supplementary Figures and Supplementary Figure Legends**

**Supplementary Figure 1. Characterization of P4 murine cerebellar NSC cultures before and after differentiation.**

**A** Heatmap: Levels of mRNA for markers of stemness (*Nanog,* *Nestin*), Hh-Gli signaling (*Gli1),* and neuronal differentiation (*Neurod1*, *Math1)* in NSCs grown in stem-cell-selective medium. Transcript levels are represented on a green-red color scale based on ΔC_t_ values. Immunofluorescence images: Representative results of NSCs staining for markers of stemness and Hh-Gli signalling (green); nuclei are counterstained with Hoechst (blue).

**B** *Left*: Levels of mRNA for genes encoding neuronal differentiation markers in NSCs grown for 48 h in differentiation medium (Diff-NSCs), as measured by RT-qPCR single assays. *P* values vs. pre-differentiation NSC controls: **P<0.01: 0.0019 (*S100 P*), 0.0083 (*Pvalb*); **P*<0.05: 0.0298 (*βIII-tubulin*), 0.0316 (*Cspg4*) (unpaired T-test). *Right:* Representative results of immunofluorescence staining of Diff-NSCs for neuronal differentiation markers (green); nuclei are counterstained with Hoechst (blue). Scale bar: 5 µm for all panels.

**Supplementary Figure 2. Functional analysis of DETs in NSCs and Diff-NSCs.**

Functional analysis with DAVID (see Table 1). Clusters are shown with heat maps indicating transcript abundance (based on normalized FPKM values) for genes belonging to each Gene Ontology (GO) category.

**Supplementary Figure 3. Expression of the Hh-signalling mediator Foxm1 in murine cerebellar development and NSCs.**

**A** Heatmap and dendrogram of the nine DETs whose genes are regulated by Hh signalling.

**B** RT-qPCR data showing Foxm1 mRNA levels in murine cerebellar tissues from postnatal days 1 (P1) to 22 (P22). *P* values vs. P1. (Mann–Whitney U test). *P<0.05: 0.0033 (P4), ****P<0.0001: 0.000086 (P15), 0.000057 (P22), NS: Not significant 0.0607 (P10).

**C** Immunoblots showing Foxm1 and α-Tubulin (loading control) in murine cerebellar tissues from postnatal days 1 (P1) to 22 (P22). Full-length immunoblots are presented in Supplementary Figure 9B.

**D** Immunofluorescence staining of endogenous Foxm1 (green) in NSCs and in Diff-NSCs. Nuclei were counterstained with Hoechst (blue). Scale bar: 5 μm.

**Supplementary Figure 4. Figures related to main Figure 2C.**

RT-qPCR data showing mRNA levels of Pcna and Casp3 in NSCs transfected with siCtrl and siFoxm1. *P* values vs siCtrl. (Mann–Whitney U test). NS: Not significant 0.065 (Pcna), 0.0713 (Casp3).

**Supplementary Figure 5. B-actin unrelated chromatin controls related to main Figures 3B and 3C.**

**A** qPCR-ChIP assay of IgG, endogenous Gli1 and Gli2 occupancy of the *Foxm1* promoter region and AcH3 in NSCs and Diff-NSCs. Eluted DNA was qPCR-amplified using primers for b-actin (Supplementary Table 7). Results are expressed as fold induction values relative to ChIP input controls. Bars represent means (SD) of three independent experiments. *P* values vs. Input (Mann-Whitney U test): **P<0.01: 0.008 (IgG, NSC), 0.00557 (IgG, Diff-NSC), 0.00631 (Gli1, NSC), 0.00604 (Gli1, Diff-NSC), 0.0045 (Gli2, NSC), 0.007 (Gli2, Diff-NSC), 0.00507 (AcH3, NSC), 0.00701 (AcH3, Diff-NSC).

**B** Luciferase activity induced in the *Foxm1* promoter region in 293T cells by Gli1, Gli2, and Mock (negative control, PCDNA). Results are normalized to pRL-CMV-Renilla luciferase (R-Luciferase). Bars represent means (SD) of at least three independent experiments, each performed in triplicate. *P* values vs. control cells (One-way ANOVA test) *Left:* *P<0.05: 0.02 (Gli1); **P<0.01: 0.005 (Gli2).

**Supplementary Figure 6. Figures related to main Figure 4.**

**A** Q-PCR single assay validation of NSCs expression of the top 15 DE miRNAs listed in Table 2. Results for each miRNA are expressed as the log_2_ fold change relative to NSC expression of the endogenous control gene *U6*. Bars represent the mean (SD) of three independent experiments. P values vs. U6 control (Mann–Whitney U test): **P<0.01: 0.0058 (miR-15b-3p), 0.0092 (miR-335-3p), 0.0069 (miR-15b-5p), 0.0074 (miR-16-2-3p), 0.0078 (miR-16-1-3p), 0.0099 (miR-15a-3p); *P<0.05: 0.029 (miR-92a-1-5p), 0.0204 (miR-130b-5p), 0.022 (miR-130a-5p), 0.031 (miR-25-5p), 0.037 (miR-93-3p), 0.042 (miR-301a-5p), 0.021 (miR-130b-3p), 0.019 (miR-106b-5p), 0.032 (miR-19a-3p).

**B** qPCR-ChIP assay of IgG, endogenous Foxm1 and AcH3 in NSCs and Diff-NSCs. Eluted DNA was qPCR-amplified using primers for b-actin (Supplementary Table 7). Results are expressed as fold induction values relative to ChIP input controls. Bars represent means (SD) of three independent experiments. *P* values vs. Input (Mann-Whitney U test): **P<0.01: 0.0068 (IgG, NSC), 0.0047 (IgG, Diff-NSC), 0.0036 (Foxm1, NSC), 0.0024 (Foxm1, Diff-NSC), 0.0091 (AcH3, NSC), 0.00448 (AcH3, Diff-NSC).

**C** RT-qPCR data showing miRNAs expression levels in NSCs transfected with siCtrl and siFoxm1. *P* values vs siCtrl. (Mann–Whitney U test). *P<0.05: 0.022 (miR-130b), 0.035 (miR-301a), 0.024 (miR-19a), 0.042 (miR-15b), 0.014(miR-15a).

**Supplementary Figure 7. Figures related to main Figure 6.**

**A** qPCR-ChIP assay of IgG, endogenous Nanog and AcH3 in NSCs and Diff-NSCs. Eluted DNA was qPCR-amplified using primers for b-actin (Supplementary Table 7). Results are expressed as fold induction values relative to ChIP input controls. Bars represent means (SD) of three independent experiments. *P* values vs. Input (Mann-Whitney U test): **P<0.01: 0.00804 (IgG, NSC), 0.0062 (IgG, Diff-NSC), 0.00336 (Nanog, NSC), 0.0052 (Nanog, Diff-NSC), 0.0085 (AcH3, NSC), 0.0034 (AcH3, Diff-NSC)

**B** Luciferase activity induced by ectopic expression of Nanog and Mock (negative control, PCDNA) in 293T cells transfected with luciferase vector carrying the wild-type Foxm1 promoter (wt) and its mutant lacking the Nanog binding sites s2 and s3 (mutants s2, s3). *P* values vs. indicated controls (Mann–Whitney U test). *P<0.05: 0.01849; ***P<0.001: 0.0002265; NS: 0.08604.

** Supplementary Figure 8.** **Full-length gels and immunoblots related to main Figure 2.**

**A** Full-length gel related to Figure 2B Left panel.

**B** Full-length immunoblot related to Figure 2B Right panel.

**C** Full-length immunoblot related to Figure 2C Left panel.

**Supplementary Figure 9. Full-length immunoblots related to main Figure 2D and Supplementary Figure 3C.**

**A** Full-length immunoblot related to Figure 2D Right panel.

**B** Full-length immunoblot related to Supplementary Figure 3C panel.

**Supplementary Table legends**

**Supplementary Table 1. Putative and validated downstream mediators of Hh-Gli signalling.**

**Supplementary Table 2. Downstream mediators of Hh signalling that are differentially transcribed in P4 murine cerebellar NSCs before and after differentiation.**

**Supplementary Table 3. Transcripts differentially expressed in NSCs and Diff-NSCs identified using the DESeq package.**

**Supplementary Table 4. Similarity matrix of human FOXM1 and murine Foxm1 promoter regions.**

**Supplementary Table 5. The 80 miRNAs displaying differential expression in NSCs vs. Diff-NSCs.**

**Supplementary Table 6. miRTarbase: Validated murine gene targets of the Foxm1-regulated miRNAs.**

**Supplementary Table 7. List of primers used for qPCR-ChIP assays.**

**Supplementary Table 8. Transcripts differentially expressed in NSCs and Diff-NSCs identified by all three methods.**

**Supplementary Table 9. List of up regulated novel isoforms of known transcripts in NSCs.**

**Supplementary Table 10. List of down regulated novel isoforms of known transcripts in NSCs.**

**Supplementary Table 11. Transcripts differentially expressed in NSCs and Diff-NSCs identified using the Cuffdiff whole-read protocol.**

**Supplementary Table 12. Transcripts differentially expressed in NSCs and Diff-NSCs identified using the Cuffdiff trimmed-read protocol.**

**Supplementary Information**

**Table of contents**

- **Supplementary Information – Section 1.** **Supplementary Methods**
- **Supplementary Information – Section 2.** **The Foxm1 promoter region.** The area analysed spanned from -3954 to the transcription start site (TSS). The 50-nt sequence upstream of the promoter is highlighted in grey. Putative binding sites for Gli (green) and for Nanog (yellow) are also shown.
- **Supplementary Information – Section 3. The miRNAs promoter regions.** Putative binding sites for Foxm1 (forward: fuchsia; reverse: dark green) are highlighted for each promoter region.
- **Supplementary Information References**

**Supplementary Information – Section 1. Supplementary Methods**

***Overview of study design***

NSCs (n=3) and Diff-NSCs (n=3) were subjected to RNA (mRNA-seq) and small-RNA (microRNA-seq) sequencing. A consensus-based approach was used to analyse the mRNA-seq dataset, in order to overcome any methodological bias inherent in specific alignment and differential expression tools. Two different tools were used for mapping and alignment, and three different approaches were used for the differential expression analysis. The intersection of all three methods resulted in common genes with similar expression changes (**Supplementary Table 8**). The expression values reported by the DESeq ^1^ algorithm were used for the remaining analysis as representative of all methods. The common genes were used as input for the pathway enrichment analysis using DAVID (david.ncifcrf.gov). Potential downstream targets of the Hedgehog (Hh) pathway were taken under further consideration (**Fig S1**).

In parallel, the microRNA-Seq dataset was analysed using the DESeq2 ^2^ algorithm to detect differential expression of microRNAs between NSCs and Diff-NSCs (**Fig S1**). Subsequently, from the list of up regulated microRNAs in NSCs we searched in the promoter region of the microRNAs for binding sites of the highest expressed gene implicated in the Hh pathway. We then validated the candidate Transcription Factors (TFs) and created the network connecting the TFs and microRNAs (**Fig S1**).

**Figure S1. Experimental and analysis design.**

***Library preparation and RNA sequencing***

*RNA extraction.* Total RNA was extracted from NSCs and Diff-NSCs using Trizol reagent (Life Technologies, USA). Quality-control assays were performed on an Agilent BioAnalyzer using the RNA Nano Kit (Agilent # 5067-1511). Only RNAs with RIN values > 9 were used for library preparation. The standard Illumina protocol and TruSeq RNA Sample Prep Kit were used to construct the mRNA-seq libraries. The mRNA in 400 ng of total RNA was converted into a library of template molecules suitable for deep sequencing analysis. In brief, we used poly-dT oligo-attached magnetic beads to purify poly-dA-containing mRNA molecules. The mRNA was then fragmented using divalent cations under elevated temperature, and the cleaved fragments were copied into first-strand cDNA using reverse transcriptase and random primers. Second-strand cDNA was then synthesized using DNA Polymerase I and RNAse H. The cDNA fragments underwent an end-repair process followed by the addition of a single ‘A’ base and ligation of the adapters. The products were purified and enriched by PCR to create the final cDNA library. All samples were sequenced using the Illumina Genome Analyzer IIx.

***Mapping and differential expression analysis of RNA-seq reads***

***Transcriptome mapping with Genomatix Mining Station and differential expression analysis with Genomatix Genome Analyzer (Method 1)***

***Mapping with Genomatix Mining Station.*** All 70mer paired-end sequenced reads were aligned to the latest version of the *Mus musculus* genome available at the time, (NCBI38/mm10), using the Genomatix Mining Station (GMS, Sesame 2.4, <https://www.genomatix.de/>). The local spliced alignment mode and point mutation algorithm were activated, where the alignment is based on pairwise comparison of nucleotides without taking into consideration insertions or deletions but point mutations only and only unique hits were selected.

We performed paired-end polyA^+^ RNA-Seq on NSCs and Diff-NSCs (three biological replicates for each cell type), with an average of 128±40 million reads per lane (**Fig S2A**). All samples were quality-controlled with the FastQC tool (<http://www.bioinformatics.babraham.ac.uk/projects/fastqc/>) before mapping, we obtained an average of 75% of reads with a quality score >30. The number of transcripts expressed in each analysed sample ranged from 168,000 to 172,000, and 80% of these were annotated in the mouse transcriptome. In terms of annotation, the read distributions for NSCs and Diff-NSCs were similar (**Fig. S2B**), confirming the absence of biases in sequencing and normalization.

***Differential expression analysis with Genomatix Genome Analyzer****.* The RNA-Seq sequenced samples were subjected to differential expression analysis using the DESeq method in the Genomatix Genome Analyzer platform (GGA, v3.30126, <https://www.genomatix.de/>). Differentially expressed transcripts were selected as those exhibiting a minimum log_2_ fold change of 1 and adj. P-value < 0.05 (P-value adjusted with multiple testing correction using the Benjamini and Hochberg method).

***Transcriptome mapping with TopHat and differential expression analysis with Cuffdiff (Methods 2 and 3)***

***Mapping of RNA-Seq reads.*** All sequenced 70mer paired-end reads were aligned to the *Mus musculus* genome (NCBI37/mm9) using the spliced read aligner TopHat version v1.3.1 ^3^. We ran two iterations of TopHat alignments to maximize the use of splice site information derived from all samples. To this end, reads from each sample were first aligned (default parameters and ‘min-anchor=5’). Next, we generated a pooled splice-site (or “junctions”) file containing all predicted splice sites across all alignments. We then re-aligned each sample using the pooled splice-site file without the option to search for new splice junctions. This strategy led to the reconstruction of 173,464 junctions, 80% of which are annotated in the University of California Santa Cruz genome browser (UCSC) ^4^*.*

***RNA-Seq transcriptome assembly.*** The transcriptome of each sample was assembled separately from the mapped reads using Cufflinks V1.2.1 ^5^ with default parameters and the UCSC reference annotation.

After performing paired-end polyA^+^ RNA-Seq, aligned reads were then assembled using the reference based transcript assembly (RABT) with the UCSC transcriptome as reference. Transcript abundance was estimated as fragments per kilobase of exon per million fragments mapped (FPKM).

Analyses of the transcript distributions after TopHat mapping and Cufflinks assembly among different samples, biological replicates and cell types exclude the presence of biases in sequencing and normalization (**Fig S3B, S3C and S3D**). The samples displayed similar proportions of alternatively spliced genes. Transcribed isoforms not annotated in the reference version of the UCSC were found in all samples (1100-1500 per sample). They are listed as putative novel isoforms in **Fig S3E** and **Supplementary Tables 9, 10**. Clustering analysis of the expression data confirmed clear separation of NSCs from their respective Diff-NSCs and the similarity of biological replicates within each group (**Fig S3F**).

The RNA-Seq reads were processed using two variations of the same protocol: the standard whole-read protocol (Cuffdiff-WR, Method 2) ^5^ as well as the variation of this protocol based on the analysis of trimmed reads (Cuffdiff-TR, Method 3). The motivation for the trimmed reads approach is based on the observation that some NSC samples had comparatively lower average base quality towards the end of the reads. By trimming all datasets to 55bp reads, we sought to increase mappability by eliminating low-quality bases, and to avoid mapping artifacts across samples by ensuring a consistent read length. Additionally, we sought to test the follow-on impact on differential expression analysis using these trimmed reads.

***RNA-Seq reads and differential expression analysis with Cuffdiff using whole reads (Method 2)***

We established an FPKM threshold to define whether a transcript is expressed using the lower bound of the expression distributions (0.3 FPKM) (**Fig S3B**). The final set included 28512 transcripts, 24210 known and 4302 putative novel isoforms of known genes, 728 genes not annotated in UCSC but overlapping with annotated genes in other databases [Ensembl ^6^ RefSeq ^7^ and NONCODE ^8^ and 1122 Novel Transcriptionally Active Regions].

Once all reads were assembled, non-expressed transcripts and transcripts not present in at least two out of the three biological replicas were removed to avoid assembly artifacts ^9^. We used Cuffcompare to produce a combined annotation file subsequently used as input for Cuffdiff along with the original alignments files produced by TopHat. Cuffdiff was used to detect differentially expressed transcripts (DETs).

***RNA-Seq differential expression analysis with Cuffdiff using trimmed reads (Method 3)***

Before mapping reads to the genome, we trimmed all reads to 55mer. Following spliced alignments using TopHat, the same pipeline and parameters described in Method 2 were used to perform differential expression analysis. As mentioned above, we sought to detect any variability in assessing differential expression using shorter reads with higher average base quality.

***Comparison of the differential expression results from all different methods***

After analysing our samples with three different methods, we selected the intersection of the results, which yielded 988 DETs (**Supplementary** **Table 8**). Comparing the expression among the three different algorithms we confirmed that the Pearson correlation between the DESeq method and Cuffdiff using whole and trimmed reads was extremely high. Specifically, for DESeq - Cuffdiff using whole reads we obtained r=0.985, p-value=0.00 and for DESeq - Cuffdiff using trimmed reads: r=0.997, p-value=0.00). Additionally, the ANOVA test was performed to verify that the values obtained using the DESeq method are representative of the other two methods (p-value=0.9567, no statistically significant differences among means and standard deviations). The Pearson correlation and ANOVA test were performed using GraphPad Prism version 6 (La Jolla, CA, USA, <http://www.graphpad.com/>). We conclude that the three approaches show a high degree of synergy and that results are not affected by methodological bias, alignment artefacts, or sequence quality. For downstream analyses, the expression values obtained from the DESeq algorithm were selected as representative of all methods.

***Functional Analysis.*** The Database for Annotation, Visualization and Integrated Discovery (DAVID, david.ncifcrf.gov) was used for functional annotation, Gene Ontology categories with a Bonferroni correction adjusted p-value < 0.05 were reported^10^.

***Clustering analysis.*** Clustering and heat maps were generated in Gene-E (version 3.0.238, <http://www.broadinstitute.org/cancer/software/GENE-E/>) (Supplementary Figure 2 for better visualization) and in R (<http://www.r-project.org/>) (the rest of the figures) using differentially expressed transcript levels as input. In Gene-E, the one minus Pearson correlation method was used for clustering and the complete linkage as a linkage method whereas in R the Bray-Curtis method and the average linkage were used in hclust to cluster the samples ^11^ and heatmap.2 to generate the heat maps.

***MicroRNA library preparation and sequencing***

Small RNA-seq libraries were prepared in accordance with Illumina's TruSeq Small RNA Sample Prep Kit protocol. In detail, total RNA was processed for an adapter ligation to each end of the RNA molecule. The RNA 3' adapter is specifically modified to target microRNAs and other small RNAs that have a 3' hydroxyl group resulting from cleavage by Dicer or other RNA processing enzymes. Reverse transcription (RT) was performed to create single-stranded cDNA. The cDNA was PCR-amplified using a common primer and a primer containing one of the 48 index sequences. The amplification product was gel-purified to generate library product of around a total 150bp. For deep sequencing analysis, - amplification with the cBot Single-End Cluster Generation Kit (Illumina) to form clonal clusters inside the flow cell. One lane of single-end 31 bp sequencing was performed for each small RNA-seq library by using the Illumina Genome Analyzer IIx.

***Mapping of microRNA-seq reads and differential expression analysis with DESeq***

***Mapping of microRNA-seq reads.*** All sequenced single-end reads (31 bases) of the three biological replicates for each cell type (NSC and Diff-NSC) were controlled with FastQC (<http://www.bioinformatics.babraham.ac.uk/projects/fastqc/)> before mapping and we obtained an average of 89% of reads with a quality score >30. All samples were aligned to the *Mus musculus* small RNA library (12-2013), (miRBase V20) using the Genomatix Mining Station (GMS, Sesame 2.4, <https://www.genomatix.de/>). To be specific deep mapping, meaning hits with up to one error tolerance and results with multiple hits were selected. Mapping to the small RNA library resulted in an average of 34±8 reads in millions for each sample. No ambiguous (match more than 50 times with equal best quality in the genome) reads were noted, 0.13% reads were ignored (no seed could be found in the index), 3.52% were insufficient (insufficient quality hits have too many mismatches to pass the specified alignment quality threshold) while 43.68% hits were unique (had exactly one best match) and 52.66% were multiple hits (had 2-50 equally best matches). The microRNA-seq read distributions for the NSCs and Diff-NSCs are shown in **Fig S5B**.

***microRNA-seq reads and differential expression analysis.*** The microRNA-seq reads were analysed for differential expression in NSCs vs. Diff-NSCs in the Genomatix Genome Analyzer (GGA, v3.30126, <https://www.genomatix.de/>) using the DESeq2 method. Differences characterized by a minimum log2 fold change of 1 and an adjusted p-value of < 0.05 (Benjamini–Hochberg correction for multiple testing) were selected for further analysis.

***Identification and characterization of binding sites in promoter regions.***

The MatInspector tool in the Genomatix Genome Analyzer was used to identify putative binding sites in the Foxm1 promoter for Gli and Nanog (**Supplementary Information – Section 2. The Foxm1 promoter region**) and putative Foxm1 binding sites in the promoters of miR-130b, miR-301a and representative members of miR-17~92 and miR-15~16 clusters (**Supplementary Information – Section 3. The miRNA promoter regions**). Occupancy of the Foxm1 promoter by endogenous Gli1, Gli2, and Nanog in NSCs and Diff-NSCs was assessed by real-time qPCR-ChIP assay. Anti-acetyl-H3 antibodies were used to identify transcriptional activation of Foxm1.

***mRNA-Seq mapping statistics***

mRNA-seq data were analysed with the Genomatix Mining Station (GMS) (Genomatix GmbH, Münich, Germany) and TopHat/Cufflinks ^5^. Mapping statistics for the two methods are summarized in **Figures S2A and S3A**, respectively. The number of reads per biological replicate that could be aligned to the mouse genome and the transcript distributions were similar with the two methods, thereby excluding the presence of biases in sequencing and normalization (**Fig S2-3**).

**Figure S2. mRNA-seq mapping statistics with Genomatix Mining Station.**

**A.** Mean (SD) number of reads successfully aligned to the mouse genome, transcriptome, and exon junctions for each NSC and Diff-NSC sample.

**B.** Average distribution of reads mapped to the genome for NSC and Diff-NSC samples.

**Figure S3. mRNA-seq mapping statistics with TopHat and Cufflinks assembly.**

**A.** Mean (SD) number of reads successfully aligned to the mouse genome, transcriptome, and exon junctions for each NSC and Diff-NSC sample.

**B.** Box plots showing FPKM values (median [IQR]) for NSCs and Diff-NSCs.

**C-D.** M-A plots for the (**C**) NSC and (**D**) Diff-NSC replicates showing the expression ratio (M) of each transcript as a function of its average abundance.

**E.** Compositions of the NSC and Diff-NSC transcriptomes.

**F.** Unsupervised hierarchical clustering of all samples using all expressed transcripts. Nodal numbers indicate bootstrap values obtained by resampling the data 10,000 times.

***Differentially expressed transcripts***

The differentially expressed transcripts (DETs) identified with the DESeq package and the Cuffdiff-WR and –TR protocols are listed in **Supplementary** **Tables 3, 11 and 12**. A total of 988 DETs were identified with all three methods (Fig. S4). Pearson correlation analysis disclosed strong correlation between the DET expression values obtained with DESeq and those generated with the other two methods (DESeq vs. Cuffdiff-WR: r=0.985, *P* value=0.00; DESeq vs. Cuffdiff-TR: r=0.997, *P* value=0.00). ANOVA confirmed that the DESeq expression values were reliably representative of the others (*P* value=0.9567), and they were therefore used for subsequent analyses of the 988 DETs.

**Figure S4 Transcripts displaying differential expression (DETs) in NSCs and Diff-NSCs.**

Venn diagram of DETs identified with DESeq, Cuffdiff-WR, and Cuffdiff-TR. (The 988 DETs identified with all three methods were analyzed as shown in **Figure 1A** and **Supplementary** **Figure 2**).

***microRNA-seq mapping statistics***

The mapping and read distribution results allowed us to proceed with the differential expression analysis without fear of bias from the sequencing and mapping (**Fig S5 and Supplementary Methods section**).

***Differentially expressed microRNAs***

Using the DESeq2 algorithm, we identified 1893 differentially expressed (DE) miRNAs, of which 80 were statistically significant. In detail, 40 miRNAs were up-regulated and 40 were down-regulated in NSC in respect to Diff-NSC (**Supplementary** **Table 5**). Hierarchical cluster of these 80 miRNAs is reported in **Figure 4A**.

**Figure S5. MiRNA-seq mapping and DE analysis statistics.**

**A.** Alignment of microRNA-seq mapping reports the Total number of reads and hits are classified as Ignored, Ambiguous, Insufficient, Multiple and Unique (Millions).

**B.** Percentages of unique, multiple and insufficient hits among microRNA-seq reads for NSCs and Diff-NSCs.

**Supplementary Information – Section 2. The Foxm1 promoter region**

Foxm1 promoter (mouse)

ttgtacaaaaaagcaggcttcgaaggagatagaaccagatcttggaattcTGGCTAGCCTGGGGCTGGCAATGTAGACCTGGCTGGCCTGGAATTCAGAGATCTGCCTGCCTCTGCCTCCTGGGTGCTGGAATTAAAGGTGTGCACCACCACACTCTGCTCACTCTTCAAACACAGTCTTATTATGTAGCCCAGACTAGAC**TAATG**T**GA**GG**CC**CTTTTGTCTCACACCAGGATTATTGGTACGTGCCACATACCTAGCTGCTTACAACTTCTAACCGGGTTCCTTCAAATATTAAATAAGAACAAAATTCAGAAAGGCGAAGACCCTGCCTCCTGTCTCATAGCATACCACACTCACTACATCTTAGGCCTTTGTATTCAGCAGGCCTCCTCCCGGTCAAAGCAGCTCTCCCTTCTCTATTTACTTCAGGGTTTCCCTCTCTTCTGT**GTAATG**G**G**TA**AC**AG**A**TCTTACACCGCGTTCTCAGCTCCTTTAAAAAAAAAAAAAGGAAAGAAAGAAAGAACGGCTTTACTGTCCTAAG**T**CAAGA**TCTCATTA**AA**A**TAAACAAAGTTATCACAGGAGGCTGCGGGAGGGCGAGCCAGT**GACCCC**GGCT**A**CTCCGAAGGCGGAAACACGAGGATCCAGAATTCTACACAACCTTGTGTCAGACAAAACCCACCGGCCTCTAACTTGTTCCTCTGTAGCTAGAATTCTACCGATC**T**GTTCA**TCTCATT**CTGTG**C**TCCCTCGCCATCAGACGCTGAGGCCAGGGGGAGGACGCCCGGGTCCGCCGCTGTATCCTCCGCTCTTATCGTAAAGTACTTCGAGGGAATAAACGATCGTCCGGACACCTCGGGGGC**TGGGG**C**CCG**GGAGGAGGCGGCCACAGACCGGAGCGGGGCGGAGCCACGTAACCGCAAGTCTAGGGCCAGAAACCCAAGC**CGGACCCCACGGAGC**CGCGTGGACTGAGCGGGGCGGGGGACCCAGGGGTAACCGAGTGGGTAGCCAGCCCGGGGAAGTGGCGTACGCTGTCCCGGAACTGCTGGCCGCCGTCCGCGCGTCCCCGCGTCCCTCCCGCCGCCCCTCGAGCCCCGCCGGGGCCGCCTTACCCGCCCTCCCGGCCGCGGCCGCCGCTCCTCAGGACACCGAGCGTTCGGGGCCGGAACCCGGAGACAAGCCGGTGCCGATTGGCGACGCTCCGTCACGTGACCGCAACGCTCCGCCGGCGCCAATTTCAAACAGCGGAACAACTGAAAGCGGCGCTGCGGGACCC**ACCCC**CGGCCCGGGCTCCCCCGTC**ACCCC**GCCCCGGGCTCCC**ACCCC**GGCCGTCCCGCCGGGACCCGCCGCCCGGGCCCGGCTCGGCCCCGCGTGGAGCAGACGCGGCCTGTGAGGTGA**GTGGGG**TGCGGCCGGGTCCAGGGAGGGCTCGGAGTCGGCACTGCCGGGGCT**GTGGGG**CGCGAGGGGGTGTTGGGCACCGCCGGGAAGAAGGTCTGAGCCTCCGGCCTCGGCCTTGCCGCCGCCGTGTTCTCCGAGCATCCCCGGACCCTGGGAACAGGCCTGCCCTCGGAGTCTGCATTTGCAGTCAACCCTGCCTCCGCCTCCACCGCTGCAGGGCGGGATCCTGGCTGAGTGTTGGTTGCAAGGTTCCCTGTTGAGTGTGGAGAATAATGGGGTCGTGGGGTGG

Provided below is the sequence (50 nt) upstream of the promoter for mouse Foxm1.

5'-ttgtacaaaaaagcaggcttcgaaggagatagaaccagatcttggaattc-3'

Putative binding sites for GLI:

5'-cggaCCCCacggagc -3’

Putative binding sites for NANOG:

5'-ggtggtAATGagacaccta-3’

3’-taggtgtctCATTaccacc-5'

**Supplementary Information – Section 3. The miRNAs promoter regions**

**Foxm1 weight matrix for putative binding sites using the Genomatix Genome Analyzer:**

5'-AACA-3'

3'-TGTT-5'

**Cluster miR-15b~16-2**

miR-16-2 promoter region

chr=3, start=69032759, end=69033359

CAGTTCTAAGCCAGGAGGAGCTTGAGGCAATCAAGAATCCAGAGTCTATAACAAATGAAATTGCACTTTTGGAAGCTCAGTGTCGTGAAATGAAACCAAACCTTGGAGCCATCGCTGAGTATAAAAAAAAGGTGTGAGTGAATTGTTTCTAGTAGAAATTTTGCTGAGCCTAAACGGGGTATTTAATTGTGCATAATGACCAGTGCTAATTCTCAGATTTGTATGTGTGTGAAAGGGTTTTGTTTTTTTGTTTTACAATCTTCACAGGAAGATTTATATTTGCAAAGAGTAGCCGAACTGGACAAAATTACTTCTGAAAGAGATAATTTTAGACAAGCATATGAAGATCTTCGAAAACAAAGGCTGAATGAATTTATGGCTGGTTTTTACGTAATAACAAATAAACTAAAAGAAAACTACCAGATGCTCACATTGGGAGGAGATGCTGAACTGGAGCTTGTGGACAGTTTAGATCCTTTTTCTGAAGGAATCATGTTCAGGTTGGTGAGATTTTAATCCTGCATCTTTTCCAAACTACTATTTCTTTAGTGTGTGAAGGTTGGAACTACATTTATTTGGTAATAACTATCTTGCTCTTACA

**miR-130a-3p promoter region**

chr=2, start=84741545, end=84742145

GGTGGGAGGAGGCTGGAGGGGGAAGCACAGAGGAAGAGCTCCCAAATGAAATTCAGGCCTCCATTTTTCCTGCTTGTTGCAATGGGGGTGGGGGGTGGTCTCCCTGAGGCTACTCCATGGTCTGAATGGATCTCCACAGGGAGGAGCCTTGCAGTCTCTGTCCCTTAGGTTAGAGCTGTAACTGGCTTCTGACCAGTATGCTTAGCTGCTGTGTGACTCTAGAGGCCTGCATGGCTCTGAGCCTTGGGTTTCAAGTGCACCTGGCTTTGGAATTTTATCAGTCTCTATTGTCCTCAATGAAATTAAGGGGGATGGCCTGGGGTCATGGAAATGAGTGTGAAGACTCCACTACTAAACCCTTGGGATAGACAGGAACCGGTGGCTTCTAAGCCCAGTGTCAATGAGAGCTTATCCTTAACTCAAGAGCCAAATCACTGGCCATTTCGCTTAATACCATATCCATAGAGTACTTTGAAGACATCAGAAAGGAAACAGTGGGGTTTCAGTTGCTTGCACCTCCAGATCTGGGAGCTTCTTTCTCCTGCCTAAGCACCTGTAGTTAGGTTCCATTATTCTAGACCTGGAGGTCACCTGAGATCAT

**Cluster miR-17~92**

chr=14, start=115045026, end=115045626

AAAGATGGCAAACTGATGGTCAGTAGAGTGACAGGTACACATGACACTCGAGTGCTGGGTCTGGAGAAGCTGCAGTTAGTATTTAGGATAACAGTATATATAATATATAGTTCTACATTTGGGCAGCACAGTTGGTTTCAGGCTATGAATAAAAATCATTGGAGTGAAACCTAAAGAAAAGTAAAATTAATAAGGAAGAGCTGCTAAAATCAGGTTTAAGCTGACACTATCTACAGAGCTAAAGTTTTCCATAATATGTTGCTTTTTTTAAAAGAAATGCAAGAAGCTGACTGATGGATCCCTGAGCTGCCAGGTAAGGATTCCACAGGCCTGGGTTTGATTGTGAGTGACCAGAATTGTACAATTATTACTAAAACAGAAACATAGTCACTTCTGACTCCATTCTTAACATTTTTGGATTAAAACTTGTTTCAGATGACTAATGAAAACTTCCTTTAAAACGTGATAAAGCTCTAATGTCAGCACGATCCACATGATCCATGTGCCTCCTATAAAGGGAGGGGTCTGTCACCAGTGTTACGGATTGAATGCTACGTTTATCTTCCAACATAGGAAGCCTGCCAGGTACTTTCTTCAATAT

**miR-335-3p promoter region**

chr=6, start=116405759, end=116406359

AAATCAAGCAGGGTCAGGTAACAGGTGAGTATATGGGCCGTGAGGTGAGTGCTGTACACAGGTCCTGGGGACTGAGGTTCTGGGGTTTAGAGCTCTGTTTCCTGAGAGAGCAGAGTAAACTACATTGAGAACACTTCCTGCTCCTTTTGGGGAGGGAGGACTTGATGAGTCAGAAATGTACAGAGTGGTTGTGGGCGTTCAGAAGCTCTGTCCTGCTTCCTTCTGACATGAGACCTCCTGATGAGCTAACTGAGTCTCCCCTGCAGGAATCCTAGAGTGGCCTTTCTGGACGAAGCTGGTAGTTGTGGCCATCGGCTTCACTGGAGGACTTCTCTTTATGTATGTTCAGTGCAAGGTGTACCTACAGTTATGGAAAAGACTCAAGGCTTACAATAGAGTGATCTATGTTCAGAACTGTCCAGAAACAAGTAAAAAGAATATTTTTGAAAAGTCTGCACTTACAGAGCCCACCCTTGAAAATAAAGAAGGACATGGAATGTGTCATTCCACCACAAATTCTTCTTGCACAGAGCCTGAAGACACTGGAGCAGAAATTATTAACGTCTGACCATGTGAGGGTGTCATTTCCTTGATGTCCACC

**Cluster miR-106b~25**

chr=5, start=138171718, end=138172922

TCTTAAGTGCCTGTCCCAACTCTACTCACCGGGAAAGCAGCTCCAGAAGGCTGAAAGGAGAGACGTTTTCTAAGGTTGTGGCCACCAAATAGAGCCCACTGTGTCTGATGTGAATAAAATGACGATCACCGTGATACTGAGGCAGAGTTGAGGACAGAGGCCATTAAGAAAAGACCAGAATTGTAGTAAGGAAACCTGCAAGATGGCACCCTGAGGTCTACTAGTGATGGGCGATGCCCCAGATCTAAGACCTGGCCGCCTAACGTGGAAAGGTAGTATCTACCTCTCAAAGTTGTTGATAGTCAAAAAATAGGGTGCATGGCTCTGTAACTGGCTTCTTGTTAGTACTCAAAACATTTATAAACAAATCCTCCCCACACACACACCCCCGCCCCCAAGCTTCCTACCAAGCTGCCCTTGACAGGGAGGAGGCACTCACCCACCTCCACAGTTCTAAGGCCCTGCACACCAACCACAGTAGTTACCATGACAACCGGGGACTCGCCTCCAGGCAGTCCCGTCAGCTTCCGGTAGAAGAGCTCTGCCACATCACGACCACCACTGTCCCCGCGGACTAGCCAGGTGGAAAGATAGTTAAGGAACAGGCGACACAGACTGATCTGGATTCGATTTCTACCCAGCTACCCTACCCCGTCTCCCGGGTCAAAGGATACAGTCTTTATAGATGAGCGGATCCCCCTTGGAAGACAGAATGAAGAACTGGGAAATCATGGCGGTTCTGGAGGATAGACGAGAACACGGTGAAAGTTGAACCCACCCCGCCTTGTGGACGCGGAACAAAAGGCCGCACGGAGGCTGGCGCTTTAAGAAACACTCCTCCCACACACACACTCGCGAGCATTAATTTCGAACCGCGGGAAACAGGGTGTCCAACCCGCCAGGATCTGGCAATCTTTCAACGGCCCGCCCTCAAGCCCCTTGTCATTCAATCTAGACTCGTCCATTGATTGGTTAAAGTTAGGGGCTGGCAGCAGTTGGGAACCCCCTATCTGATTGGCGGGTTCTCAGCCGCACGGGTCACATGGGTGACGACGTTTCGCGCCAATTTCGGTTGGCCGGCTACGTCCCGCCGCGCGTTCGTTTTCTGCTTCCCCAGAGAGAGATTTTTGAGCCCTTCAAGTCCTGCCACACCGTCCCCGGCAGCGATGGCGCTTAAGGACTACGCGATCGAAAAAGGTGAAGAT

**miR-130b promoter region**

chr=16, start=17121616, end=17122216

AATATAGCTAGAATTACATAGGAGACCTTGTCTCAAAAAACAAGCAAAAGAAGTGGTCAGGACATGATCTGGAGAGGGATGTGTGTAATGCTTCCGAGTAGGTAGGAAACTCAGAGGGTGCAAAAGCCAGAGCTAAAGCCCTTTGGGGGGCAGAAGGAAGTCCTTTTGGATATTATCTTAGTGAACTGGAAAGTAGATTCAATTACCAGGGCGGTCTGGTAGGGCAGAGTATGGAGGGAACTGCAAACTGGGAGAGGGCTGGGTGCTTTGAAACGGAAAGGACCACGGCTCAGAAGGAGGTCAAAATCCAGGAGCTCAAAATCCAGAAGGTACCTGGGTGCCTTTGGCGGTCACAGTGAGGGGTGGAGCAGTGGGGGCGGGGTGGGGTGGGGAGCACACCAATGCGCATGCCTAGAGAAGTGAGCATTGTTACGCGTACTGGTGCTGGACCCGCCCCCTGTCTCTGTGGGCATTCATTCCCTGGGGTAGACCGGGAGGGGGGTGGGTAGTCTGGGGATCTGGAGTCTGCACTGCGGCTGTAGGGAGAGAGTGGGTACGCCCTGCAGTCCTCATCCCGATGGTTTCCCATCACCTTTCTCAG

**miR-301a promoter region**

chr=11, start=86926083, end=86926683

TTTTCATTTGTTTTTTGAAGTAATTACTCAAAGGACTTGTTTTCTATGGTAGGTAACCCAGACGCTTTGGAAAAGGAGTTAACCAACATTACATTTAGGCAAAAAACATAGTTTTTCACATATTTGCCTTAGTAGTAATTACAGCTGATTTTGTTAATATCAATTATAAAGTTTTTATGATGCCCATGTTAGCTACTAAAGGCTCAGGACATATTACAAAATTCCTTTACATCTGTGCATGTTTATGTCTGAATGCTGCTAATTTCAGGTTAGTCAACAAAAACCAATTATCACTTTGGTCCTTTGCCCTTCCAAAAATATACTGGTATAAATGTTTTTAGGTTTTGGATGTCTTCTGACTAAAATGGCAACTCAACTTTAAGGTCTTTGCACACTGAGCCTTGCATTCCTTTCCTGCTCACTCCTGCTAACGGCTGCTCTGACTTTATTGCACTACTGTACTTTACAGCGAGCAGTGCAATAGTATTGTCAAAGCATCCGCGAGCAGGTTGCACACCTTTCCTGGTTCTTGTCTTACATCTTCAGTATTTGTGAAAAGGTTTCTATAACTTTCAGTGTGGAAAACTGTATAATTTTGTTA

**cluster miR-15a~16-1 promoter region**

miR-15a promoter region

chr= 14, start= 61619651, end= 61620251

CCTATAGTTTTGGCATTTGAATGGTAGAGTTGGCAGAATGAGAAGTTCAATTTGTCTTCAGCTATATAAGGTTGACATTAAGCTAGCCTATAGAGAACCCCATACCCCCAAAAGAAAAAGTCTTTGTTTTATAAGTTGTGGCCTACCAACTTTCAAAGTATAGGACACGATGCATTCTCAGATATCCTGGTTTATAGTAGTTTTGTTTTTTGTTTGTTTGCTCCTTGAGGCAGGGTCTTTGTAGTACTGCCTGGCCTGGGACTCACTATGCTGAAAAAGCTGTCCTGGAATTCATGAAGTCTTCTAGCAAGGAAGTTGGACGTGATTATTTTTGTCTCCTAAGAGTAAAGAATATCTAAATCAAATAGCAATAAGAATGGAACTGATCCTGAAGCAAAGTGATACCTGCGTGTATCCTAGCCATTGGGAGGTGAAGGAAGGAAGTGGGAAACAGCATATCAAGGTAAAAAACTTGGAACATTATTAAGATAGAAAGGAGGTATAAGGAGCCCGTGCTCTTGTTCTGGAAGGTGACGGTGATGTTACTGAAGGATGAGAAACCACCTGAAACACTGAGCCTTCTCACAATATTACATTTGAA

**Supplementary Information References**

1 Anders, S. & Huber, W. Differential expression of RNA-Seq data at the gene level–the DESeq package. *Heidelberg, Germany: European Molecular Biology Laboratory (EMBL)* (2012).

2 Love, M. I., Huber, W. & Anders, S. Moderated estimation of fold change and dispersion for RNA-seq data with DESeq2. *Genome biology* **15**, 550 (2014).

3 Trapnell, C., Pachter, L. & Salzberg, S. L. TopHat: discovering splice junctions with RNA-Seq. *Bioinformatics* **25**, 1105-1111 (2009).

4 Meyer, L. R. *et al.* The UCSC Genome Browser database: extensions and updates 2013. *Nucleic acids research* **41**, D64-D69 (2013).

5 Trapnell, C. *et al.* Differential gene and transcript expression analysis of RNA-seq experiments with TopHat and Cufflinks. *Nature protocols* **7**, 562-578 (2012).

6 Flicek, P. *et al.* Ensembl 2013. *Nucleic acids research*, gks1236 (2012).

7 Pruitt, K. D., Tatusova, T. & Maglott, D. R. NCBI reference sequences (RefSeq): a curated non-redundant sequence database of genomes, transcripts and proteins. *Nucleic acids research* **35**, D61-D65 (2007).

8 Bu, D. *et al.* NONCODE v3. 0: integrative annotation of long noncoding RNAs. *Nucleic acids research*, gkr1175 (2011).

9 Prensner, J. R. *et al.* Transcriptome sequencing across a prostate cancer cohort identifies PCAT-1, an unannotated lincRNA implicated in disease progression. *Nature biotechnology* **29**, 742-749 (2011).

10 Huang, D. W., Sherman, B. T. & Lempicki, R. A. Systematic and integrative analysis of large gene lists using DAVID bioinformatics resources. *Nature protocols* **4**, 44-57 (2009).

11 Suzuki, R. & Shimodaira, H. Pvclust: an R package for assessing the uncertainty in hierarchical clustering. *Bioinformatics* **22**, 1540-1542 (2006).
